# Differential Effects of a Novel Opioid Ligand UTA1003 on Antinociceptive Tolerance and Motor Behaviour

**DOI:** 10.3390/ph15070789

**Published:** 2022-06-24

**Authors:** Alok K. Paul, Krystel L. Woolley, Mohammed Rahmatullah, Polrat Wilairatana, Jason A. Smith, Nuri Gueven, Nikolas Dietis

**Affiliations:** 1 School of Pharmacy and Pharmacology, University of Tasmania, Hobart, TAS 7001, Australia; alok.paul@utas.edu.au (A.K.P.); nuri.guven@utas.edu.au (N.G.); 2 School of Natural Sciences, University of Tasmania, Hobart, TAS 7001, Australia; krystel.woolley@utas.edu.au (K.L.W.); jason.smith@utas.edu.au (J.A.S.); 3 Department of Biotechnology & Genetic Engineering, University of Development Alternative, Lalmatia, Dhaka 1207, Bangladesh; rahmatm@uoda.edu.bd; 4 Department of Clinical Tropical Medicine, Faculty of Tropical Medicine, Mahidol University, Bangkok 10400, Thailand; 5 Medical School, University of Cyprus, Nicosia 1678, Cyprus

**Keywords:** UTA1003, UFP-505, morphine, antinociception, antinociceptive tolerance, motor behaviour, mixed opioid, mixed activity

## Abstract

Analgesic tolerance is a major problem in the clinic for the maintenance of opioid-induced long-term pain relief. Opioids with mixed activity on multiple opioid receptors promise reduced antinociceptive tolerance in preclinical studies, but these compounds typically show poor bioavailability upon oral, subcutaneous, intraperitoneal, or intravenous administration. We designed UTA1003 as a novel opioid that acts as a mu (MOP) and kappa (KOP) opioid receptor agonist and a partial agonist for delta (DOP) opioid receptor. In the present study, its antinociceptive effects, as well as its effects on antinociceptive tolerance and motor behaviour, were investigated in male rats. Acute antinociception was measured before (basal) and at different time points after subcutaneous injection of UTA1003 or morphine using the tail flick and hot plate assays. Various motor behavioural activities, including horizontal locomotion, rearing, and turning, were automatically measured in an open-field arena. The antinociceptive and behavioural effects of repeated administration of UTA1003 and morphine were determined over eight days. UTA1003 induced mild antinociceptive effects after acute administration but induced no tolerance after repeated treatment. Importantly, UTA1003 co-treatment with morphine prevented antinociceptive tolerance compared to morphine alone. UTA1003 showed less motor suppression than morphine in both acute and sub-chronic treatment regimens, while it did not affect morphine-induced motor suppression or hyper-excitation. Based on these activities, we speculate that UTA1003 crosses the blood-brain barrier after subcutaneous administration and, therefore, could be developed as a lead molecule to avoid opioid-induced antinociceptive tolerance and motor suppression. Further structural modifications to improve its antinociceptive effects, toxicity profile, and ADME parameters are nevertheless required.

## 1. Introduction

Opioids, especially morphine and other µ-opioid receptor (MOP) agonists, are widely used drugs for the clinical treatment of chronic pain [1,2,3]. However, long-term repeated use of clinical opioids such as morphine is often limited by their liabilities to induce a significant range of side effects, such as respiratory depression, sedation, constipation, addiction, dependence, withdrawal symptoms, behavioural suppression, and analgesic tolerance [4,5]. Tolerance is a phenomenon of reduced efficacy of an effect of a drug over repeated use of a particular dose [6]. Studies showed that there are differences in onset or magnitude of tolerance to antinociceptive and other behavioural effects, such as sedation, nausea, ventilatory or respiratory depression [7,8,9]. Antinociceptive effects and tolerance of morphine are both dependent on dose, dosing frequency, and duration of treatment [10]. In contrast, morphine-induced behavioural side effects in the clinic are both reported as dose-dependent (e.g., pruritus) and dose-independent (e.g., nausea, vomiting) [11,12,13]. Antinociceptive and behavioural adverse effects of morphine are also dependent on ageing and age-dependent changes in absorption, distribution, metabolism, and elimination (ADME) capabilities [14]. At present, the collective evidence suggests that antinociceptive tolerance to morphine does not necessarily translate to tolerance against behavioural side effects.

Therefore, a number of studies investigated different ways to prevent antinociceptive tolerance to morphine and its behavioural adverse effects. A combination of a MOP receptor agonist (e.g., morphine) and a delta opioid (DOP) receptor antagonist (e.g., naltrindole, naltrexone) has previously shown better antinociception and less tolerance than an MOP receptor agonist alone [15,16,17,18,19,20]. These studies and others investigating different combinations of multifunctionality increased the interest in identifying single ligands that have mixed selectivity for different opioid receptors (bifunctional ligands), as these compounds have shown a potential for reduced tolerance, sufficient antinociception, and possibly a better pharmacokinetic profile than a combination of two individual drugs [21,22,23]. As a result, MOP and DOP receptor-selective opioid ligands with mixed activity (MOP receptor agonist/DOP receptor antagonist; MOP/DOP receptor agonists) have been described with reduced antinociceptive tolerance and physical dependence or reward responses compared to clinically used opioids such as morphine or fentanyl [22,24,25,26,27,28,29,30,31]. These results indicate that MOP and DOP receptor interactions are essential to manage antinociceptive tolerance, although the detailed mechanisms underlying this effect are not understood. On the other hand, MOP/KOP receptor agonists reduce cocaine abuse after co-administration [21,32], while MOP/NOP agonist ligands have been reported as non-addictive analgesics that are effective in treating neuropathic pain [33,34]. Nevertheless, the profile of many new opioids and their antinociceptive tolerance induction after repeated treatment has not been reported previously, which significantly hinders the detailed interpretation of experimental data and their clinical development.

**Figure 1:**
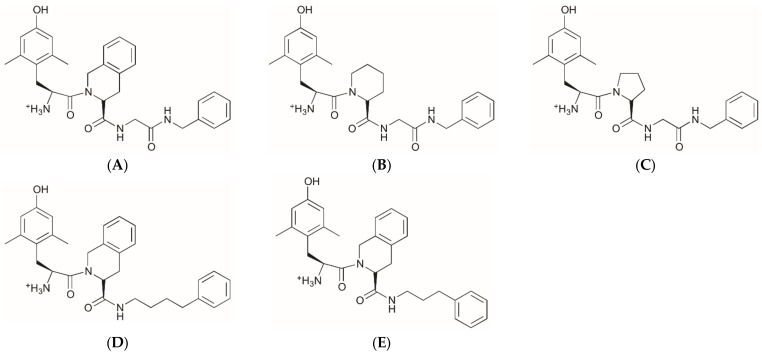
Chemical structures of UFP-505 and four UTA-opioids. (**A**) UFP-505; (**B**) UTA1003; (**C**) UTA1005; (**D**) UTA1009; (**E**) UTA1011.

Opioids with mixed activity on MOP and DOP receptors, such as UFP-505 (H-Dmt-Tic-Gly-NH-Bzl), were reported as MOP receptor agonists and partial agonist/antagonist efficacy at DOP receptor depending on receptor expression [35]. UFP-505 produced antinociception after intracerebroventricular (i.c.v.) and intrathecal (i.t.) administrations in rodents, but it induced antinociceptive tolerance and behavioural toxicity after repeated treatment [35,36]. Importantly, UFP-505 did not appear to induce its antinociceptive effects after subcutaneous administration [35]. Chemical analogues of UFP-505 with better solubility can be administered systematically and might show an improved pharmacological profile, potent antinociceptive effects, and reduced toxicity with preclinical treatment. As a part of ongoing investigations to identify opioids with selectivity for multiple receptors, we describe four novel UTA-opioids based on UFP-505 (Figure 1). Measurement of G-protein activation (or blockage) following a ligand binding to a G-protein-coupled receptor (GTPγ^35^S binding assay) or measurement of its effects in downstream pathways, such as ligand binding-induced changes in cellular cAMP levels (cAMP assay), are typically used to characterise novel opioids [25,30,37,38]. We used cAMP mobilisation as a tool to characterise the specificity of UTA ligands using MOP, DOP, KOP, or NOP receptor-expressing CHO cells and selected the ligand UTA1003 with a MOP receptor agonist/DOP receptor partial agonist profile out of four UTA-opioids for subsequent preclinical studies. We hypothesised that UTA1003 might induce less antinociceptive tolerance and behavioural side effects than morphine. We also speculated that after co-administration with morphine over several days, UTA1003 might also reduce antinociceptive tolerance and behavioural side effects of morphine. Therefore, the current study investigated the motor behavioural side effects, antinociception, and antinociceptive tolerance after repeated administration of the novel peptide UTA1003, morphine, and their combination. To the best of our knowledge, this study, for the first time, describes whether the novel ligand UTA1003 is a potential drug candidate to induce antinociception with reduced adverse effects compared to morphine.

## 2. Results

### 2.1. Structures of Novel UTA-Opioids

In this study, analogues of UFP-505 (Figure 1A) were designed and synthesised with the objective of increasing their solubility sequentially and reducing their peptidic structure (Figure 1B–E). These chemical changes were hypothesised to improve ADME (absorption, distribution, metabolism, and elimination) characteristics and metabolic stability of the compounds while retaining their antinociceptive effects.

### 2.2. Specificity of Novel UTA-Opioids on Different Opioid Receptors

**Figure 2:**
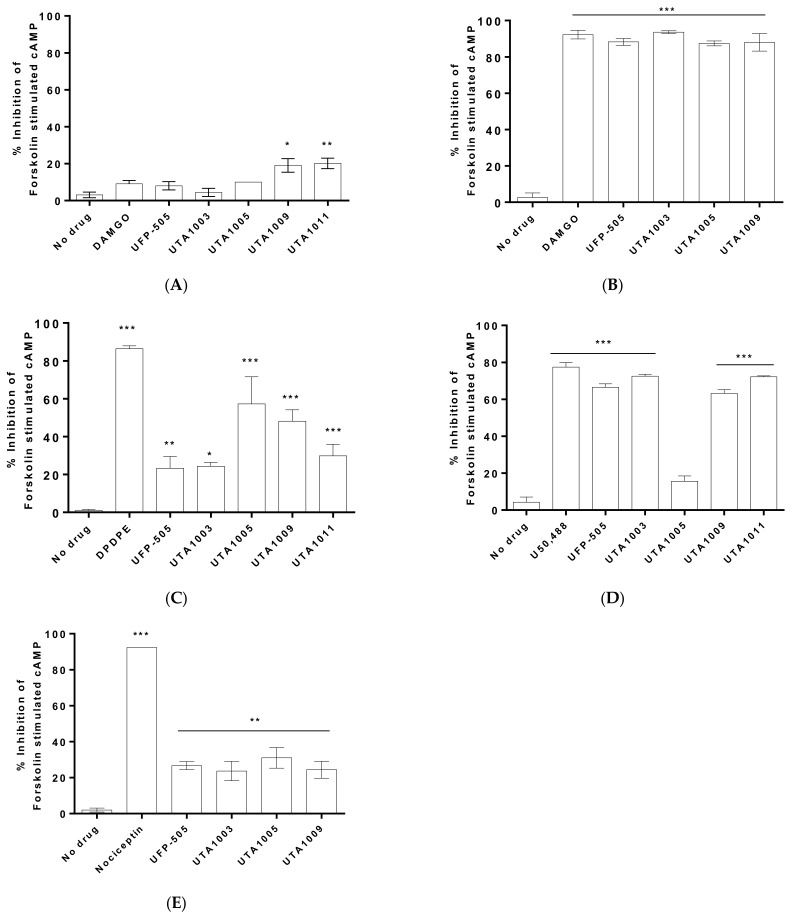
Effects of UTA-opioids on wild-type (**A**), µ-opioid receptor (**B**), δ-opioid receptor (**C**), κ-opioid receptor (**D**), nociceptin/orphanin-FQ receptor (**E**) expressing Chinese hamster ovary (CHO) cells. Inhibition of forskolin-stimulated cAMP levels was measured in the absence or presence of 100 µM UTA and reference opioids. Statistically significant differences against the effect of 1 µM forskolin (no drug) is expressed as * (*p* < 0.01), ** (*p* < 0.001), or *** (*p* < 0.0001) and were calculated using one-way ANOVA with Bonferroni’s multiple comparisons test. Values represent the mean ± SEM (n = 6 wells per group). Error bars are present in all graphs but are sometimes too small to be visible.

The specificity of the four novel ligands for different opioid receptors was measured using several recombinant human opioid receptors expressing Chinese hamster ovary (CHO) cell lines. Firstly, the ligands were evaluated in untransfected Chinese hamster ovary (CHO-WT) cells to confirm that the UTA ligand-mediated effects are opioid receptor specific (Figure 2A). Using this assay, UTA1009 and UTA1011 had a slight but significant (*p* < 0.01) inhibitory effect on forskolin-stimulated cAMP production (Figure 2A). This effect was not observed for UTA1003, UTA1005, UFP-505, and DAMGO.

To investigate whether the novel UTA-opioids are MOP receptor agonists, all ligands were measured for their specificity in MOP receptor-expressing Chinese hamster ovary (CHO-MOP) cells (Figure 2B). All opioids significantly (one-way ANOVA, F (7, 36) = 368, *p* < 0.0001) inhibited forskolin-stimulated cAMP production (Figure 2B), and therefore, these ligands acted as MOP receptor agonists. Notably, all responses were comparable to those of the reference compounds UFP-505 and DAMGO (Figure 2B).

Since UFP-505 is described as a MOP receptor agonist/DOP receptor antagonist, we assessed whether the novel UTA ligands showed any affinity towards the DOP receptor (Figure 2C). Both UFP-505 and UTA1003 showed significantly lower specificity than DPDPE (a DOP receptor agonist) (one-way ANOVA, F (8, 32) = 52.09, *p* < 0.0001) (Figure 2C) and induced identical effects with regard to the DOP receptor. The remaining three UTA-opioids showed significant inhibition of forskolin-induced cAMP production (one-way ANOVA, F (8, 32) = 52.09, *p* < 0.0001), which was similar to the effects of DPDPE (Figure 2C).

UFP-505 was described as a partial agonist (pK_i_ 6.29) of the KOP receptor in a previous report [37]. In this study, the specificity profile of UTA-opioids was measured using CHO-KOP cells with U50,488 (a KOP receptor agonist) as a reference compound (Figure 2D). Most ligands showed specificity for the KOP receptor except UTA1005 (Figure 2D).

UFP-505 is known to be a partial agonist of the NOP receptor with a pK_i_ of 5.86 [37]. In this study, all UTA ligands were assessed for their effects on the non-opioid receptor (NOP receptor) using CHO-NOP cells (Figure 2E). In this experiment, all opioids significantly inhibited forskolin-stimulated cAMP levels. However, their efficacy was significantly lower than the reference compound, nociceptin (a NOP receptor agonist) (one-way ANOVA, F (8, 32) = 52.09, *p* < 0.0001) (Figure 2E).

### 2.3. Detailed Agonistic Effects of UTA1003 towards Different Opioid Receptors

From the specificity profile assessment undertaken for this study, UTA1009 and UTA1011 are categorised as non-selective, as these ligands induced their effects on wild-type cells. Moreover, UTA1005 appeared to be full agonists of both MOP and DOP receptors. Based on the specificity profiles of these opioids, UTA1003 was chosen as the most suitable ligand for further pharmacological characterisation, as it likely represented a MOP receptor agonist/DOP receptor partial agonist that promised the desired characteristics. Subsequently, EC_50_ values for UTA1003 and reference compounds (DAMGO, DPDPE, U50,488, and nociceptin) were determined from the dose-response curves for different opioid receptors.

**Figure 3:**
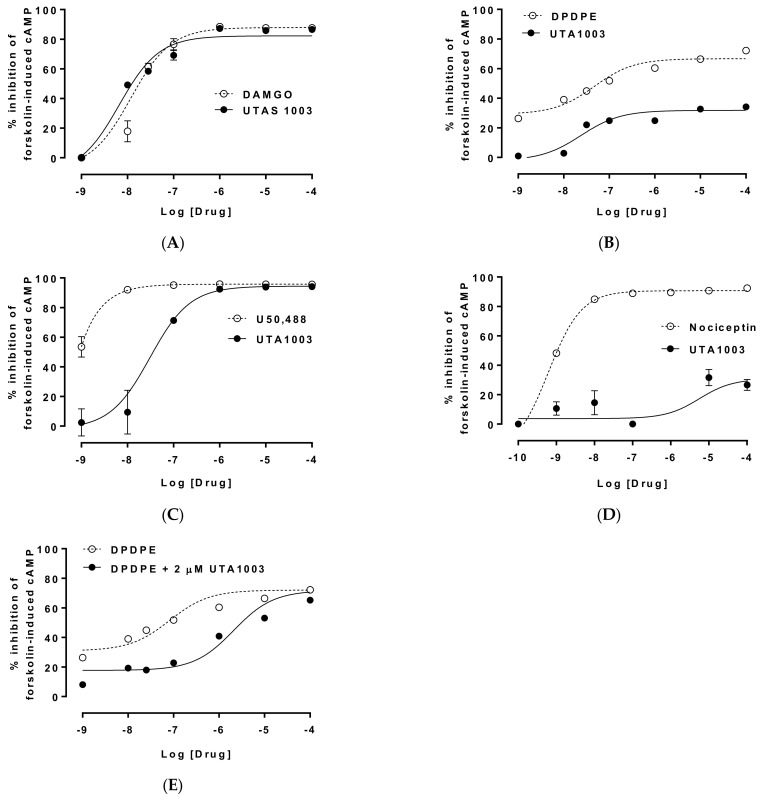
Dose-response of the agonistic effects of UTA1003 on different opioid receptors and antagonistic effect against the effect of DPDPE on DOP receptor. The MOP, DOP, KOP, and NOP receptor agonistic effects of UTA1003 were assessed as inhibition (%) of forskolin-stimulated cAMP levels in CHO cells expressing human MOP, DOP, KOP, or NOP receptor, respectively (**A**–**D**). The cAMP was stimulated using 1 µM (5 µM for KOP and NOP) forskolin. DAMGO (1 nM–100 µM), DPDPE (1 nM–100 µM), U50,488 (1 nM–100 µM) or nociceptin (0.1 nM–100 µM) was used as positive control. Different molar concentrations (M) of DPDPE (1 nM–100 µM) vs. a combination of 2 µM UTA1003 and DPDPE used for the assessment of the antagonism of UTA1003 in human DOP receptor-expressing CHO cells (**E**). Values are presented as mean ± SEM (n = 6 wells per group). Error bars are sometimes too small to be visualised.

In this study, the efficacy of UTA1003 for the MOP receptor was determined from a dose-response curve in CHO-MOP cells, where UTA1003 appeared to display similar effects to DAMGO, as their pEC_50_ and E_max_ values were indistinguishable (Figure 3A, Table 1). We determined the efficacy (as EC_50_) of UTA1003 in cells expressing the DOP receptor, and a parallel dose-response curve and EC_50_ were determined for the selective DOP receptor agonist, DPDPE (Figure 3B). The pEC_50_ value of UTA1003 was similar to DPDPE (Table 1), but the magnitude of the efficacy (E_max_) of UTA1003 was lower than the E_max_ of DPDPE (Figure 3B). Therefore, UTA1003 appears to be a partial agonist for the DOP receptor.

During the screening test with CHO-KOP cells, 100 µM UTA1003 induced a similar agonistic effect compared to U50,488 (Figure 2D). To investigate the magnitude of agonism of UTA1003 towards the KOP receptor, UTA1003 was further assessed in CHO-KOP cells (Figure 3C). Although UTA1003 at higher concentration showed similar efficacy to U50,488, the agonistic activity of this ligand is noticeably weaker than U50,488 (Figure 3C, Table 1). Although UTA1003 showed significantly lower agonistic effects than nociceptin at high concentrations (100 µM) (Figure 2E), we further investigated the agonistic effect of UTA1003 (0.1 nM to 100 µM) in CHO-NOP cells (Figure 3D). UTA1003 showed partial agonism (EC50 1.79 µM) towards the NOP receptor. However, this effect was much lower compared to the results of the full agonist nociceptin (EC_50_ 0.69 nM) (Figure 3D, Table 1).

### 2.4. Antagonistic Effect of UTA1003 on DOP Receptors

A previous study found that some compounds that demonstrate low agonistic activity towards a particular receptor can also exhibit antagonistic effects [39]. Since UTA1003 appeared to be a partial agonist (E_max_ 28.2%) of the DOP receptor (Figure 2E), we further investigated its potential antagonism of this receptor using CHO-DOP cells (Figure 3E). The antagonist effect of UTA1003 was calculated as described in previous studies [25,37,38]. The percentage (%) inhibition of forskolin-stimulated cAMP production was calculated for different concentrations of DPDPE with or without 2 µM UTA1003 in DOP receptor-expressing Chinese hamster ovary (CHO-DOP) cells (Figure 3E). Since the addition of UTA1003 (in DPDPE solution) shifted the dose-response curve of DPDPE (dose range: 10 nM to 10 µM) towards the right, expressing the characteristics of a DOP receptor antagonist (Figure 3E). The K_d_ value of UTA1003 was calculated as K_d_ = 47.8 nM using the formula, K_d_ = [UTA1003]/(CR − 1), where CR is the ratio of the EC_50_ of DPDPE in the presence and absence of UTA1003 [25,36,38] (Figure 3E, Table 1).

### 2.5. Toxicity of UTA1003

In the present study, we did not observe any physiological or behavioural toxicity in our animals after subcutaneous administration of UTA1003 and UFP-505. Repeated morphine dosing is known to induce itching [40,41,42], which was absent in the UTA1003-treated animals in this study. It is an important advancement as a previous study reported behavioural toxicity for two MOP/NOP receptor agonists after subcutaneous administration [33].

### 2.6. Antinociceptive Effect of UTA1003 after Acute Administration

**Figure 4:**
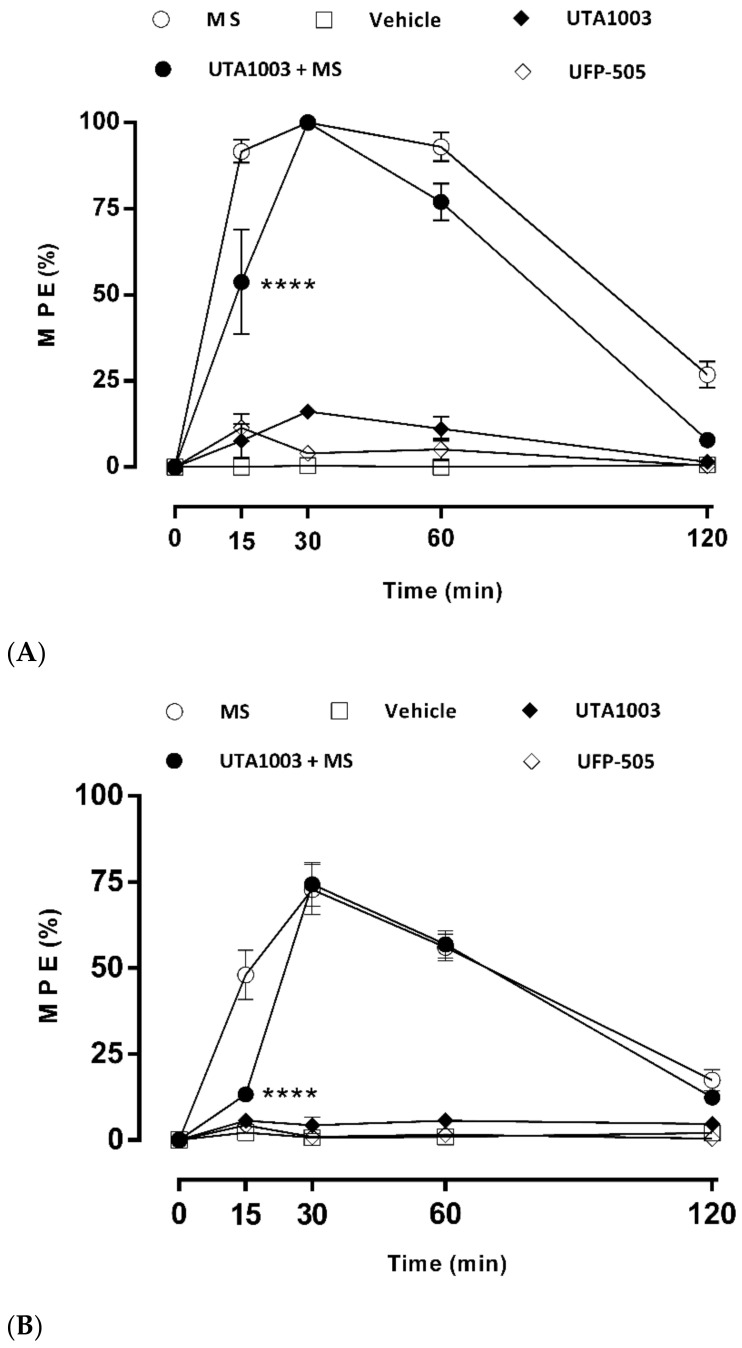
Antinociceptive effects of different opioids after acute treatment. Antinociceptive effects after single subcutaneous injections of UFP-505 (27.1 mg/kg), UTA1003 (24.6 mg/kg), morphine (MS, 3.0 mg/kg) or vehicle were measured in Sprague Dawley rats. Antinociception was measured over a period of 120 min using tail flick (**A**) and hot plate (**B**) assays. Statistical significance against the effect of MS is shown as **** *p* < 0.0001 for the same time point and was calculated using one-way ANOVA with Bonferroni’s multiple comparisons. Values are presented as mean ± SEM (n = 6 animals per group).

Two opioids with mixed selectivity on different opioid receptors, UFP-505 and UTA1003, were evaluated for their antinociceptive effects after acute subcutaneous injections using male Sprague Dawley rats on day 1. Their antinociceptive effects were compared to morphine or vehicle on day 1. Before opioid administration, no significant differences in nociceptive levels were observed in the five different treatment groups using both the tail flick and hot plate antinociception tests (Figure 4). It indicates that none of the animals used in this study demonstrated hyperalgesia or increased pain sensitivity. Baseline antinociception measurement is essential from a clinical perspective, as people experience hyperalgesia after long-term treatment with low-dose opioids [43,44]. Hyperalgesia has also been observed in preclinical studies, although the exact mechanism by which it occurs is not completely understood [45,46]. Previous studies show that hyperalgesia is not mediated by the brain MOP receptor and is also not associated with plasma concentration of morphine-3-glucuronide but rather a consequence of protein kinase C gamma (PKCγ) and NMDA receptor subtype NR1 upregulation in the spinal cord [47,48]. A 2-way ANOVA-repeated measure analysis showed significant interactions of post-administration time as F (4, 20) = 96.53; *p* < 0.0001), group of animals as F (4, 20) = 133; *p* < 0.0001, and both factors as F (16, 80) = 33.11; *p* < 0.0001). Vehicle-treated animals did not show any antinociceptive effects or hypersensitivity in both antinociception tests over a period of 120 min after administration (Figure 4A,B). However, 15 min post administration, morphine-induced significant antinociception compared to vehicle-treated animals (one-way ANOVA; F (24, 122) = 87.8; *p* < 0.0001) before it decreased gradually until 120 min (one-way ANOVA; F (24, 122) = 87.8; *p* < 0.001) (Figure 4A,B). UTA1003 and UTA1001 did not show any effects in both assays when one- or two-way ANOVA was used to compare the results among all groups. However, UTA1003 induced significant antinociception 15 min post injection in the hot plate assay (unpaired *t*-test; t (9) = 2.53; *p* < 0.05) and from 30 min onwards in both assays (unpaired *t*-test; t (5) = 4.544; *p* < 0.01 (tail flick); t (7) = 3.35; *p* < 0.05 (hot plate)) (Figure 4). The antinociceptive effect of UTA1003 was significantly different from the effects of vehicle until 60 min (unpaired *t*-test; t (9) = 4.08; *p* < 0.01) using hot plate (Figure 4B) and 120 min (unpaired *t*-test; t (9) = 2.483; *p* < 0.05) using tail flick assay (Figure 4A), respectively.

The combination of UTA1003 and morphine-induced significant antinociception from 15 min post administration (one-way ANOVA; F (24, 122) = 87.77; *p* < 0.0001) in the tail flick assay while antinociception peaked at 30 min in both antinociceptive assays (one-way ANOVA; F (24, 122) = 87.77; *p* < 0.0001 (tail flick); one-way ANOVA; F (24, 118) = 67.07; *p* < 0.0001) (Figure 4A,B). Subsequently, the antinociceptive effect of this drug combination gradually declined over the 120 min observation period (Figure 4). However, the antinociceptive effect of the UTA1003/morphine combination produced significantly less antinociception than morphine alone at 15 min (tail flick: one-way ANOVA; F (24, 122) = 87.77; *p* < 0.0001; hot plate: one-way ANOVA; F (24, 118) = 67.07; *p* < 0.0001) post administration (Figure 4). In comparison, over the 2 h observation period UFP-505 only showed some minor but statistically significant antinociception at 15 min post injection (unpaired *t*-test; t (7) = 3.18; *p* < 0.05), which was not evident using one-way ANOVA analysis (Figure 4A,B). Antinociceptive latency (in seconds) of the data shown in Figure 4 are presented in Supplementary Figure S1.

### 2.7. Effect of UTA1003 on Morphine-Induced Antinociceptive Tolerance

**Figure 5:**
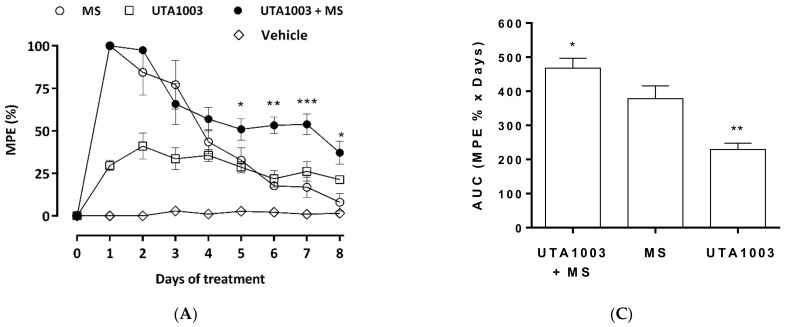

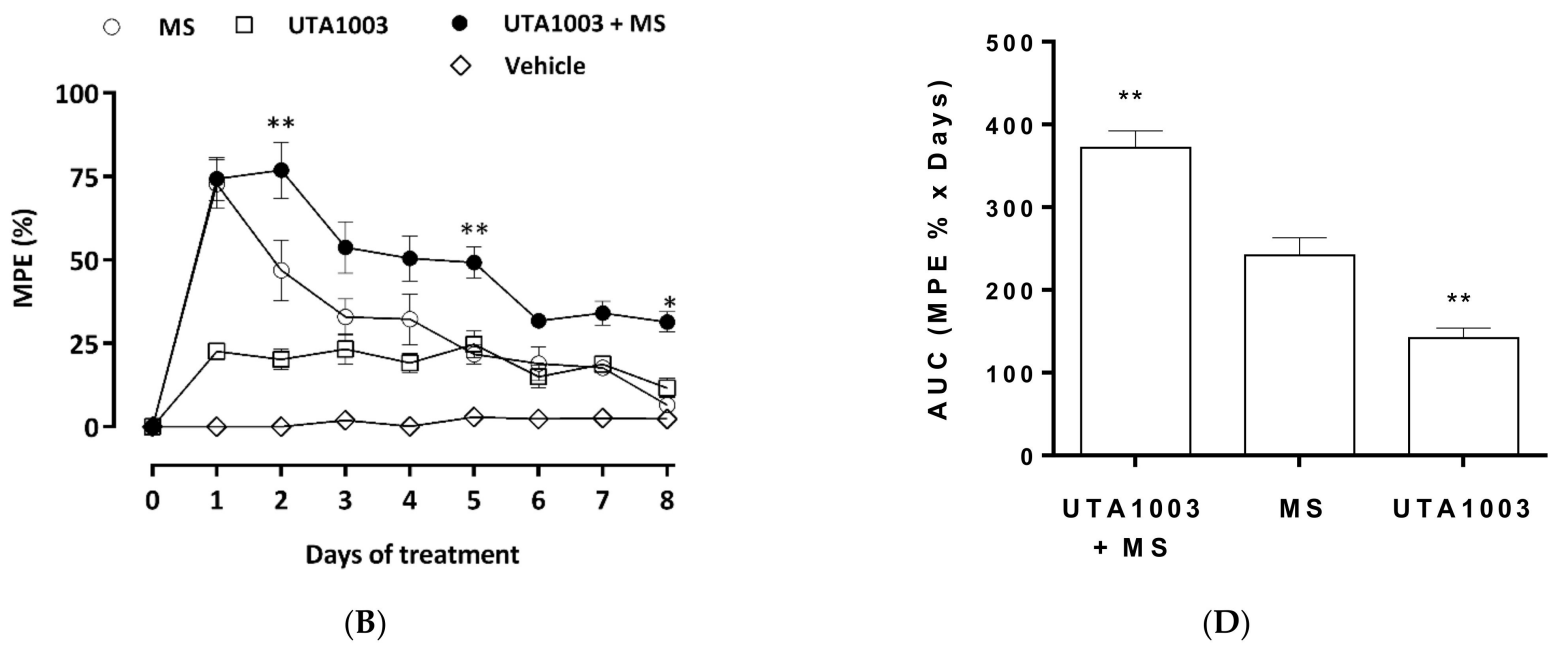
Antinociceptive effects of chronic administration of opioids. Antinociceptive effects after twice-daily subcutaneous treatment with vehicle, UTA1003 (24.6 mg/kg), morphine (MS, 3 mg/kg), or their combination were measured in Sprague Dawley rats. Antinociception was measured daily at 30 min post injections over a period of 8 days using tail flick (**A**) and hot plate (**B**) assays. Statistically significant differences in antinociception at the same time point were calculated between MS and MS + UTA1003-treated animals. Total antinociception was calculated using area under the curve analysis (AUC) for the tail flick (**C**) and hot plate (**D**) assays. Statistically significant differences in total antinociception were calculated for each group against MS-treated animals. Significance was calculated using one-way ANOVA with Bonferroni’s multiple comparisons and shown as * *p* < 0.05, ** *p* < 0.005, *** *p* < 0.0001. Values are presented as mean ± SEM (n = 6 animals per group).

To investigate the effects of UTA1003 on antinociceptive tolerance and its interaction with morphine, opioids were administered individually or in combination twice daily over a period of 8 days. Antinociceptive effects of these opioids were measured daily using tail flick and hot plate tests 30 min post administration. Antinociceptive effects of UTA1003 were 27 ± 3.73% and 22.57 ± 1.77% MPE, respectively, in the tail flick and hot plate assays on day 1 (Figure 5A,B). However, there was no significant reduction (tolerance) or increment (hyperalgesia) of antinociception observed in this group of animals throughout the 8 days observation period (Figure 5A,B). Therefore, UTA1003-induced antinociception (% MPE) on day 8 was 20.52 ± 2.13% and 15.47 ± 2.55%, respectively, for tail flick and hot plate assays (Figure 5A,B).

On the other hand, morphine produced complete antinociception (100% MPE) on day 1 in the tail flick assay. The combined treatment of UTA1003 and morphine also showed similar antinociception levels comparable to morphine itself on day 1 (Figure 5A,B). Two-way ANOVA analysis showed significant interactions of post-treatment days of repeated treatment as F (7, 83) = 27.53; *p* < 0.0001), treatment groups as F (2, 83) = 47.65; *p* < 0.0001, and both factors as F (14, 83) = 5.79; *p* < 0.0001). The full antinociceptive effects of morphine were maintained until day 2, and then a statistically significant reduction was observed on day 4 (one-way ANOVA; F (26, 101) = 25.47; *p* < 0.0001) using tail flick assay (Figure 5A). Beyond that, morphine-induced antinociception gradually declined over the observation period (Figure 5A). In contrast, the UTA1003/morphine-treated animals showed tolerance from day 3 (one-way ANOVA; F (26, 101) = 25.47; *p* < 0.0001) and the effect was (51.94 ± 4.46%) (Figure 5A). These co-treated animals maintained the antinociceptive effects of the drug combination until day 8 (43.17 ± 4.45%), with no statistical differences between days 3 and 8 (Figure 5A). Noticeably, the combination of UTA1003/morphine-induced significantly higher antinociception level on day 7 (one-way ANOVA; F (26, 101) = 25.47; *p* < 0.05), compared to morphine-treated animals (Figure 5A). Area under the curves analysis of tail flick antinociception showed that AUCs of morphine was higher than UTA1003 (one-way ANOVA; F (2, 13) = 16.77; *p* < 0.01); but lower than UTA1003/morphine group (one-way ANOVA; F (2, 13) = 16.77; *p* < 0.05).

The antinociception of morphine and the combination of UTA1003/morphine were further investigated using the hot plate assay where both groups showed similar antinociception on day 1 (72.77 ± 7.30% and 74.28 ± 6.41% MPE, respectively) (Figure 5B). Two-way ANOVA analysis showed significant interaction of post-treatment days of repeated treatment as F (8, 117) = 40.52; *p* < 0.0001), treatment groups as F (2, 117) = 74.37; *p* < 0.0001, and both factors as F (16, 117) = 5.89; *p* < 0.0001). Morphine-treated animals showed antinociceptive tolerance from day 3 (one-way ANOVA; F (26, 120) = 22.19; *p* < 0.0001) to day 8 (one-way ANOVA; F (26, 120) = 22.19; *p* < 0.0001) (Figure 5B). UTA1003/morphine did not show signs of antinociceptive tolerance until day 3 (one-way ANOVA; F (26, 120) = 22.19; *p* < 0.01). Beyond that, a 50% antinociceptive effect was maintained until day 5 (Figure 5B). This effect further decreased over time until the end of our observation period (Figure 5B). Noticeably, the antinociceptive effects of morphine were significantly lower than those of the UTA1003/morphine co-treatment on day 2 (one-way ANOVA; F (17, 80) = 20.30; *p* < 0.01), day 5 (one-way ANOVA; F (17, 80) = 20.30; *p* < 0.01), and day 8 (one-way ANOVA; F (17, 80) = 20.30; *p* < 0.05) (Figure 5B). Arear under the curves analysis of hot plate antinociception showed that AUCs of morphine was higher than UTA1003 (one-way ANOVA; F (2, 15) = 38.1; *p* < 0.01); but lower than UTA1003/morphine group (one-way ANOVA; F (2, 15) = 38.1; *p* < 0.01). Antinociceptive latency (in seconds) of Figure 5A and Figure 5B are presented as Supplementary Figure S2A and S2B, respectively.

### 2.8. Effect of Acute Administration of UTA1003 on Motor Behaviour

To gain a basic understanding of the behavioural effects of UTA1003, motor behaviour was analysed using an open-field paradigm over a period of 120 min after drug administration. These effects were compared to vehicle- and morphine-treated animals. The behavioural parameters assessed in the open-field arena were divided into three major categories: locomotion, rotation, and rearing.

**Figure 6:**
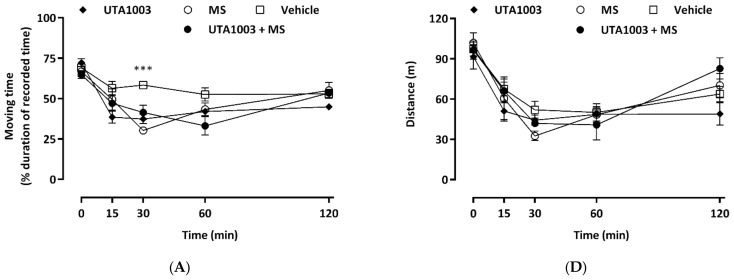

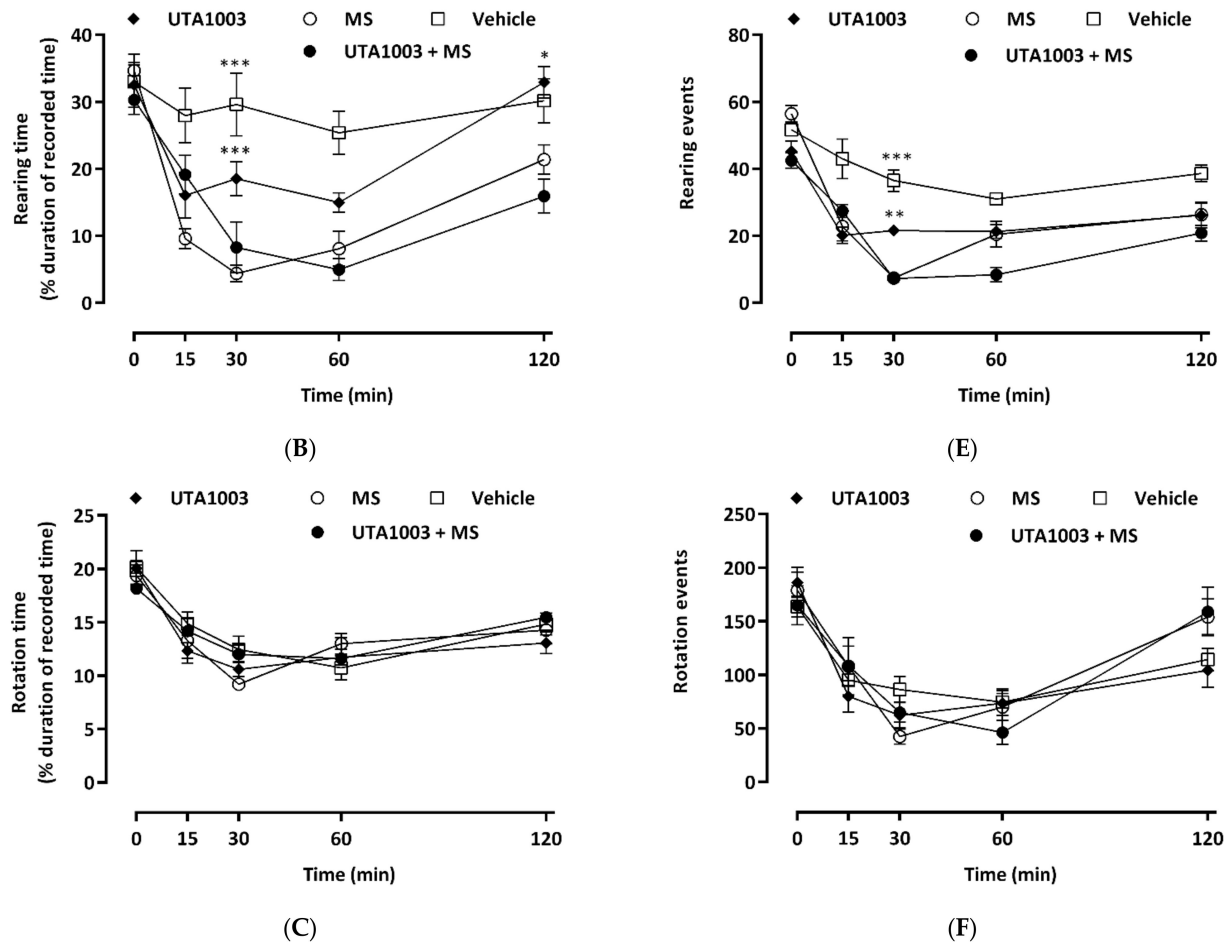
Acute time-dependent effects of morphine and UTA1003 on motor behaviour. Open-field locomotor activities after acute subcutaneous injections of UTA1003 (24.6 mg/kg), morphine (MS, 3.0 mg/kg), their combination, or vehicle were measured in Sprague Dawley rats. The behaviour of treated animals was measured as moving time (**A**), rearing time (**B**), rotation time (**C**), distance travelled (**D**), rearing numbers (**E**), or rotation numbers (**F**) over a period of 120 min. Statistical significance against morphine is shown as * *p* < 0.05, ** *p* < 0.01 and *** *p* < 0.001 at the same time point and was calculated using one-way ANOVA with Bonferroni’s multiple comparisons. Values are presented as mean ± SEM (n = 6 animals per group).

The locomotion parameter was further subdivided into the parameters of moving time and distance to gain a more detailed picture of the drug-induced effects (Figure 6A,D). Before the administration of opioids or vehicle, no behavioural differences were observed between the animal groups (Figure 6A,D). After acute administration of UTA1003, significantly reduced moving time compared to the vehicle group at 30 min post administration (one-way ANOVA; F (9, 38) = 20.97; *p* < 0.001), while moving distance was unaffected (Figure 6).

Rearing is a complex behaviour controlled by the hippocampus [49,50]. It is an exploratory behaviour that can be affected by anxiety or escape behaviour and can also increase as a result of opioid withdrawal symptoms [49,50,51,52]. UTA1003 produced slightly reduced rearing time and rearing numbers than vehicle but it was significantly higher than morphine’s suppression of rearing at 30 min post-administration time (rearing number: one-way ANOVA; F (10, 47) = 24.12; *p* < 0.001; rearing time: one-way ANOVA; F (9, 41) = 19.05; *p* < 0.001) (Figure 6B,E).

To investigate these opioid-induced effects on spontaneous behaviour further, rotation or turning behaviour was measured, which is generally believed to be an indicator of movement coordination [53]. In the present study, no differences were observed between clockwise and counter-clockwise rotations for both vehicle- or morphine-treated animals (data not shown). Similarly, total rotational activities (sum-total of clockwise and counter-clockwise rotation numbers or time) remained unaffected by UTA1003 over the entire observation period (Figure 6C,F).

**Figure 7:**
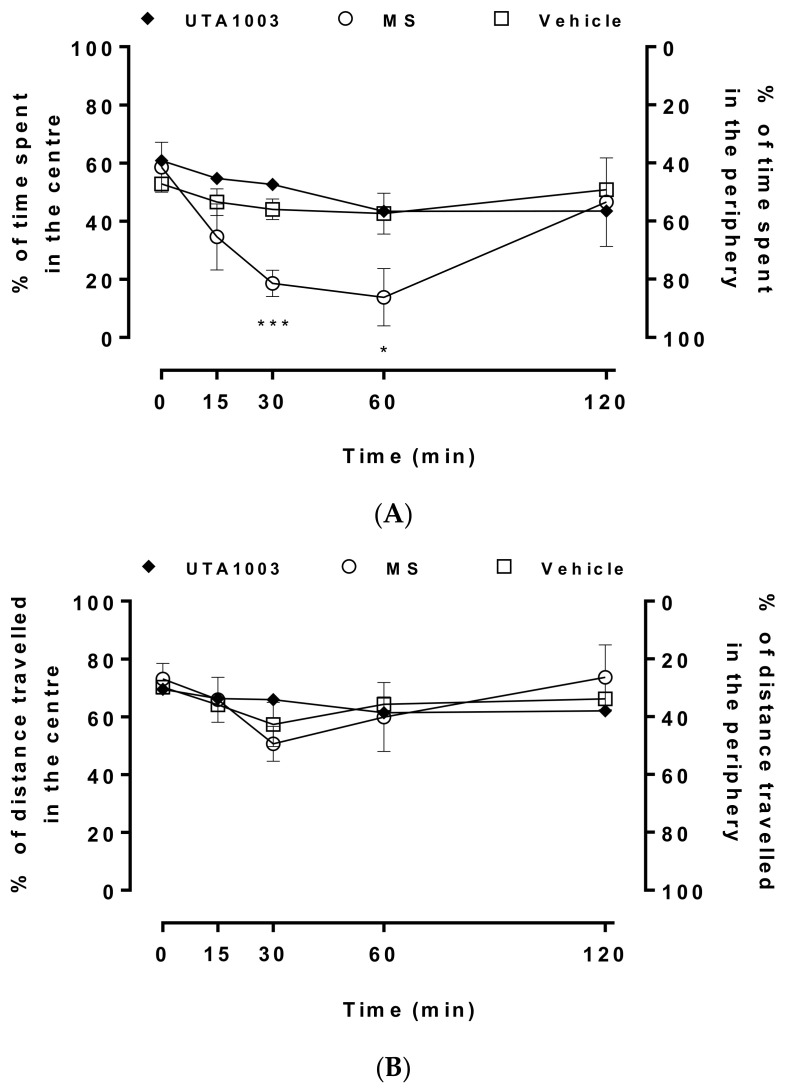
Place preferences after acute administration of opioids. Effects on time spent in the centre (% of total observation period, 5 min) or periphery (**A**) and distance travelled in the centre (% of total distance travelled by the animal) or periphery (**B**) of an open-field arena over a period of 120 min, after a single subcutaneous injection of vehicle or morphine (MS, 3.0 mg/kg) or UTA1003 (24.6 mg/kg) or UTA1003 (24.6 mg/kg) plus morphine (3.0 mg/kg), in Sprague Dawley rats. Statistically significant differences in a time point compared to vehicle in every group of animals are indicated (* *p* < 0.05, *** *p* < 0.001) and were calculated using one-way ANOVA with a Bonferroni’s multiple comparison test. Values are presented as mean ± SEM (n = 6 animals per group).

The motor behaviour of these animals was further investigated to identify the prevalence of anxiety among these animals (Figure 7). Place preferences in the open-field arena are a behavioural marker for anxiety and locomotion [54,55]. In the current study, vehicle-, UTA1003- and UTA1003/morphine-treated animals showed no difference with regards to basal place preferences over the entire observation period (Figure 7). On the other hand, morphine-treated rats spent significantly less time in centre between 30 (one-way ANOVA; F (4, 23) = 7.38; *p* < 0.01) and 60 min (one-way ANOVA; F (4, 23) = 7.38; *p* < 0.05) after administration (Figure 7A). No difference in travelled distance was observed among the vehicle-, UTA1003- and UTA1003/morphine-treated animals (Figure 7B).

### 2.9. Effect of Chronic UTA1003 Treatment on Motor Behaviour

**Figure 8:**
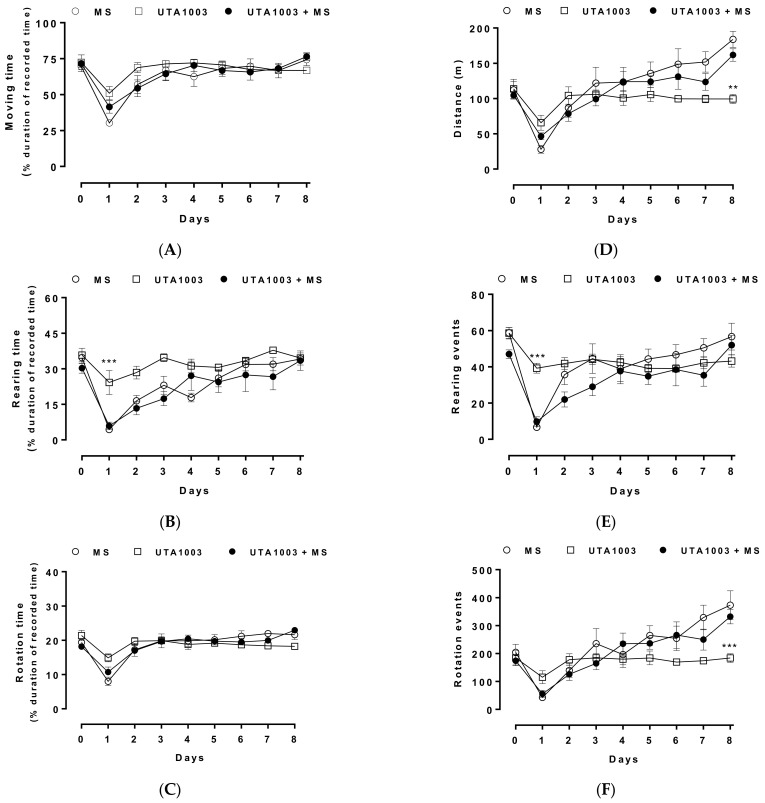
Motor behavioural effects of chronic administration of morphine and UTA1003. Open-field motor behavioural effects after twice-daily subcutaneous injections of UTA1003 (24.6 mg/kg), morphine (MS, 3.0 mg/kg), or their combination were measured in Sprague Dawley rats. Opioid-induced behavioural activities were measured daily at 30 min post injection over a period of 8 days, as described in the methods. Several behavioural parameters, such as moving time (**A**), rearing time (**B**), rotation time (**C**), distance travelled (**D**), rearing numbers (**E**), or rotation numbers (**F**), were measured. Statistically significant differences for the same time point between the effects of MS and UTA1003 are shown as ** *p* < 0.01 and *** *p* < 0.001 were calculated using one-way ANOVA with Bonferroni’s multiple comparisons. Values are presented as mean ± SEM (n = 6 animals per group).

In addition to the acute effects of opioid treatment on behaviour, motor behaviour was also determined during long-term treatment with UTA1003. In this study, animals were treated with morphine, UTA1003, or the combination of UTA1003/morphine on a twice-daily dosage regimen over a period of eight consecutive days. Motor behaviour was measured daily as locomotion, rotation, and rearing activities at 30 min post administration using an open-field arena (Figure 8).

To compare the overall motor behavioural activities among different groups of animals (as shown in Figure 8), the effect of morphine was first compared against UTA1003 or UTA1003/morphine groups. No differences were noticed between morphine and UTA1003/morphine groups over the whole observation period, but distance travelled (one-way ANOVA; F (26, 113) = 9.53; *p* < 0.01) and rotation numbers (one-way ANOVA; F (26, 95) = 10.81; *p* < 0.001) of morphine-treated animals were higher than UTA1003 group only on day 8.

## 3. Discussion

Long-term treatment with opioids is associated with a loss of therapeutic potential, analgesic tolerance, and various types of behavioural side effects. Currently, all clinically used analgesics are MOP receptor agonists [1,2,3], while their adverse effects are also transmitted by this receptor [56,57,58]. Novel mixed activity ligands, especially with MOP and DOP receptor selectivity, promise less antinociceptive tolerance than conventional, clinically used opioids [22,24,25,26,27,28,29,30,31]. This study described a novel opioid (UTA1003), a structural analogue based on the reference compound UFP-505 [37,59], with which it shares MOP receptor agonist/DOP receptor partial agonist selectivity in vitro but shows higher solubility than UFP-505.

Several compounds based on “Dmt-Tic”-containing peptides (e.g., UFP-505) display MOP receptor agonist/DOP receptor antagonist or partial agonist profiles [60,61,62,63]. It is thought that the “Dmt-Tic” moiety of UFP-505 is required for DOP receptor antagonism, while the Bzl (benzylamine) and spacer (Gly-NH) moieties are required for MOP receptor agonism [60,64]. Deleting the benzene ring from the “Tic” peptide of UFP-505 produced UTA1003, which subjectively displayed better solubility in line with a significantly reduced logP value (−0.11 versus 0.41), although detailed solubility experiments were not performed. This structural change resulted in comparable MOP receptor agonism with slightly reduced DOP receptor antagonism (pK_b_: 7.32) on compared to UFP-505 (pK_b_: 9.81 [37]; 10.50 [60]). The dose-dependent efficacy of UTA1003 towards the MOP receptor was similar to DAMGO, but its efficacy towards DOP and NOP receptors was substantially lower than the reference compounds DPDPE and nociceptin. The detailed dose-response analysis indicates that UTA1003 is also a KOP receptor agonist/NOP receptor partial agonist, similar to UFP-505 [37]. Therefore, deletion of the benzene ring from the “Tic” pharmacophore only had a minor impact on the pharmacological efficacy of “Dmt-Tic” peptides apart from increased efficacy towards the KOP receptor.

Previous studies hypothesised that the “Gly-NH-Bzl” peptide moiety is essential for the MOP receptor agonist activity of peptide opioid ligands [37,60,64]. In contrast, the replacement of glycine (Gly-NH) with a hydrocarbon chain in UTA1009 and UTA1011 retained their agonistic activity towards the MOP receptor. Since this structural change generated non-selective compounds that acted on both opioid and non-opioid receptors, the “Gly-NH” moiety appears to be essential to maintaining opioid receptor specificity overall. We acknowledge that broad concentration ranges for every ligand would better substantiate our conclusions, but these experiments only aimed to generate the prerequisite data to assess the more relevant antinociceptive and adverse effects of UTA1003 in vivo.

In this study, morphine produced maximum antinociception 30 min after injection, as described previously [10]. UTA1003 showed a very mild and similar antinociceptive response like UFP-505, while previous reports did not describe any antinociceptive activity of UFP-505 [59]. In the present study, repeated morphine treatment induced antinociceptive tolerance from days 3–4, comparable to previous studies [10,31]. Importantly, the combination of UTA1003/morphine reduced antinociceptive tolerance of morphine, comparable to previous studies that used morphine in combination with a DOP antagonist [15,16,17,18,20]. The UTA1003/morphine combination did not prevent morphine tolerance on day 3 (in the tail flick assay) but maintained nearly 50% antinociception levels over the remainder of the 8 days period. This 50% antinociceptive effect was statistically significant compared to basal antinociception (day 0). Consequently, drugs such as UTA1003 could be developed as a co-treatment with clinically used opioids, irrespective of their intrinsicly low antinociceptive activities.

UTA1003 appears to be only a weak antinociceptive compound after subcutaneous administration (which was also dependent on analytical methods) in comparison to morphine. This could be a consequence of its peptidic structure, as peptides are known to poorly cross the blood-brain barrier (BBB) [65]. However, UTA1003 suppressed motor behaviour to some extent, similar to morphine. Moreover, an approximately five times lower dose of UTA1003 reduced oxidative damage in the rat hippocampus, which suggests that UTA1003 can penetrate the BBB [66]. This interpretation supports a previous report where several other opioid peptides (e.g., Dmt-DALDA, ADAMB, and MZ-2) crossed the BBB after systemic administration in animals [67].

UTA1003 induced significant levels of antinociception between 30 and 120 min post administration in the tail flick test, in contrast to the hot plate assay, where it showed the highest efficacy 15 to 60 min post-injection. This difference could indicate that the antinociceptive effects of UTA1003 are preferentially supra-spinally mediated, as the tail flick test measures predominantly spinal-mediated nociception, while the hot plate assay mostly measures supra-spinal-mediated nociception [68,69,70]. Two different assays were deliberately employed to exclude bias when assessing antinociception since the tail flick assay reportedly overestimates morphine-induced antinociception [69]. Additionally, a comparatively low dose of morphine (3 mg/kg) was used to induce antinociception [71,72,73,74,75] to avoid the tail flick assay-based overestimation of morphine-induced antinociception [69]. The present study showed that the combination of UTA1003 and morphine prevented antinociceptive tolerance when using the hot plate assay. Morphine uniformly distributes within the brain stem and spinal cord after systemic administration, and therefore its antinociception is mainly mediated by a spinal response [76,77,78,79], while our data suggest that the antinociception of UTA1003 is supra-spinally mediated. Therefore, the combination of morphine and UTA1003 more effectively reduces peripherally mediated than centrally mediated antinociceptive tolerance. Future studies will test this hypothesis using methylnaltrexone to block peripherally mediated opioid effects without affecting centrally mediated effects [27,80].

Behavioural effects can be measured as changes to motor behaviour, movement coordination, exploratory behaviour, naloxone-precipitated withdrawal symptoms, and reward or drug-seeking behaviour [22,27,81,82,83,84]. The present study assessed motor behaviour simultaneously with antinociception in the same animals as described [81,85,86,87,88]. Morphine produced biphasic effects on locomotor activities after both acute and repeated administration, as previously reported [81,82,86,88]. Repeated treatment with morphine or the morphine/UTA1003 combination initially produced similar levels of hypo-activity with subsequent recovery (tolerance) and hyperactivity as described [88,89,90]. In contrast, UTA1003 showed only a mild but significant suppression of motor activity on the first day that returned to basal levels over the 8 days observation period. Surprisingly, co-treatment with UTA1003/morphine prevented morphine-induced antinociceptive tolerance but did not affect the morphine-induced changes to locomotor behaviour. Since both opioidergic and dopaminergic neurotransmission systems control locomotor activities [84], we speculate that UTA1003-mediated effects are mostly regulated by opioidergic neurons, with negligible effects on dopaminergic neurons, although future studies will have to validate this hypothesis.

Rearing activity is thought to be a reliable parameter to assess exploratory behaviour [50,85,91], which is why the suppressed rearing in response to repeated morphine exposure in the present study indicates reduced exploratory behaviour, as reported previously [92]. This effect was interpreted to reflect morphine-induced clinical sedation or drowsiness [84,88,93,94]. Morphine-induced reduced rearing could also be a result of drug-induced anxiety-like or depressive-like behaviour. However, this is in stark contrast to previous studies that reported both anxiolytic [95,96] and anti-depressant effects of morphine [97,98,99]. Therefore, our data cannot determine if the lack of rearing of morphine-treated rats was a result of reduced exploratory behaviour or anxiety.

As previously reported, morphine also reduced turning behaviour [100,101], which is a complex behaviour that is mainly mediated by the dopaminergic system [102,103,104]. The observed morphine-dependent suppression of turning behaviour replicates two earlier studies that reported similar levels of suppression [100,101]. Morphine-induced rotation is also subject to tolerance after repeated administration (Figure 8), which follows a similar pattern to morphine-induced antinociceptive tolerance (Figure 5).

Although the described effects of morphine and UTA1003 on behaviour appear significant and replicate previous studies, our results raise significant concerns. This study observed significantly reduced moving distance, rearing, and rotation 30–120 min after exposure to the vehicle (10% DMSO, 90% saline). These surprising results support a previous study that reported similar effects after intracerebroventricular (i.c.v.) and oral administration of DMSO in mice, although information related to subcutaneous treatment was not reported [105]. The vehicle-induced reduction in motor activities in the present study could represent anxiety-related behaviour as repeated open-field measurements can induce time-dependent behavioural changes [106]. Decreased locomotion in the central area of the open-field indicates anxiety-like behaviour [54,107,108,109,110], but since the vehicle-treated animals of this study did not show any changes in locomotion in the central area of the open-field over the whole observation period, vehicle-mediated effects are unlikely a sign of anxiety [105,111,112]. This effect requires to be investigated by future studies using specialised behavioural paradigms, while at present, it has to be seen as a major confounding factor.

Overall, UTA1003 produced no antinociceptive tolerance and prevented, to some extent, the morphine-induced antinociceptive tolerance after repeated administration. UTA1003 also induced less motor suppression than morphine, although it did not interfere with morphine-induced suppression or hyper-excitation of motor behaviour. Therefore, subsequent studies will focus on detailed preclinical pharmacokinetics of UTA1003 and its structural analogues to increase their systemic efficacy. UTA1003 represents a promising lead compound with an advantageous toxicity profile to develop potent analgesics with reduced adverse effects.

## 4. Materials and Methods

### 4.1. Materials

Cellular cAMP levels were measured in CHO cells with recombinant expression of different types of human opioid receptors. Wild-type CHO cells without recombinant opioid receptor expression were also used to investigate whether the measured effects are associated with specific opioid receptors. The Promega cAMP-Glo^TM^ Max assay kit (V1681, Promega Corporation, Madison, WI, USA) was used in this study. The commercial kit contained cAMP 100 mM cAMP, 1 M MgCl_2_, cAMP-Glo^TM^ ONE-buffer, Protein Kinase A, Kinase-Glo^®^ lyophilised substrate, and Kinase-Glo^®^ buffer. In addition, IBMX (3,7-Dihydro-1-methyl-3-(2-methylpropyl)-1*H*-purine-2,6-dione) and Ro20-1724 (4-(3-Butoxy-4-methoxyphenyl)methyl-2-imidazolidone), Forskolin ([3*R*-(3α,4aβ,5β,6β,6aα,10α,10aβ,10bα)]-5-(acetyloxy)-3-ethenyldodecahydro-6,10,10b-trihydroxy-3,4a,7,7,10a-pentamethyl-1*H*-naphtho[2,1-*b*]pyran-1-one) were purchased from Tocris Bioscience, Bristol, UK. IBMX and Ro20-1724 are phosphodiesterase inhibitors to prevent hydrolysis of cAMP [113]. Forskolin, as a positive control, activates adenylyl cyclase enzymes and increases intracellular levels of cAMP [114]. IBMX and Ro20-1724 were diluted to 100 mM solution using 100% DMSO. The solution was further diluted to 500 µM IBMX and 100 µM Ro20-1724 using PBS. Afterwards, a complete induction buffer was prepared using 500 µM IBMX and 100 µM Ro20-1724 and 25 mM MgCl_2_. The solution was used to see the basal cAMP expression of cells in the wells without treatment. Forskolin was diluted to 200 µM using DMSO, which was further diluted to 1–50 µM with freshly prepared complete induction buffer immediately before the experiments. Forskolin was used as a positive control, and all other UTA ligands and standard compounds were diluted in forskolin solution. The cAMP detection solution was freshly prepared using a 1:100 ratio of PKA and cAMP-Glo buffer (as stated in the supplier’s guide). Kinase-Glo^®^ buffer and substrate were mixed, and it was called as Kinase-Glo reagent. The Kinase-Glo reagent was stored at −20 °C as 1 mL aliquots. The required amount of Kinase-Glo reagent aliquots was thawed at room temperature and used immediately. UTA ligands were dissolved at 10 mM with 100% DMSO, and the solution was further diluted to the required concentrations using diluted forskolin solution. Dimethyl sulfoxide (DMSO) and sodium chloride were purchased from Sigma-Aldrich. Morphine sulfate was obtained as a 30 mg/mL stock solution (Hameln Pharmaceuticals GmbH, Hamelin, Germany). The analogues of UFP-505 have been synthesised as a drug optimisation study and described in a previous report [115]. Stock solutions and final dilutions of these drugs were prepared under aseptic conditions using 10% DMSO in 0.9% sodium chloride solution as a vehicle.

### 4.2. Chemical Synthesis

UFP505 was synthesised by the method previously reported [60]. Novel compounds UTA1001, UTA1005, UTA1009, and UTA1011 were synthesised similarly to the method for UFP505, and details are provided in the Supplementary Materials. By-products were analysed by mass spectrometry (please refer to the Supplementary Materials).

### 4.3. cAMP Assay

**Figure 9:**
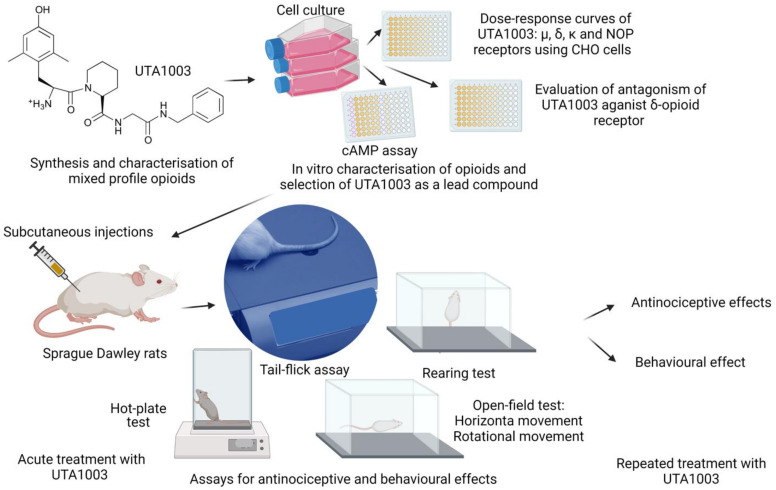
A schematic diagram of the methodologies used for evaluating UTA1003. The Figure was made with www.biorender.com (accessed on: 19 June 2022).

CHO cells were grown in T-25 flasks containing F-12 (Ham) media (1×) for the wild-type, MOP, DOP, KOP, and NOP cells containing 10% fetal bovine serum and 100 IU/mL penicillin (Figure 9). All media contained L-glutamine. The cell culture media were additionally supplemented with 200 mg/mL G418 (selection agent used with MOP, DOP, and KOP cells) and with an additional 200 mg/mL hygromycin B for the NOP cells once in every four passages. Cell cultures were kept at 37 °C 5% *v*/*v* CO_2_ and 95% *v*/*v* humidified air. Trypsin/EDTA was used as the minimum required to split the cells during sub-cultures. The cells were used for experiments as they approached confluence.

The assay was conducted using the supplier’s protocol. In short, the CHO cells were seeded as 8000–10,000 cells/well in 96-well plates and incubated for 24 h. On the following day, all reagents were thawed and prepared as per the supplier’s guidelines. Firstly, after removing media using an aspirator, cells were incubated in 40 µL of complete induction buffer (blank), no drug (forskolin 1–50 µM), UTA-opioids, and reference drugs for 20 min at 37 °C (incubator). Secondly, 10 µL of freshly prepared cAMP detection solution was added to all wells and mixed by a plate shaker for 2 min. The plate was further incubated for 20 min at room temperature (22–23 °C). Afterwards, 50 µL Kinase-Glo reagent was added to each well and mixed by a plate shaker for a minute, and the plate was incubated for 10 min at room temperature. Finally, 60 to 80 µL solutions from each well were transferred carefully to a white round bottom plate and immediately put into a plate-reader to measure luminescence. The measurement was repeated twice (after 1 min of the first measurement) to verify the luminescence signals. Each drug was tested using six different wells, and the average value was used for statistical analysis.

For a direct comparison between the UTA ligands, standard compounds, and for the concentration-response curves, the pharmacological effect was calculated as % of inhibition of forskolin-induced cAMP production, and the values were used for statistical analysis. Antagonist effects of UTA1003 were calculated as follows, K_d_ = [opioid]/(CR − 1), where CR is the ratio of the EC_50_ of DPDPE in the presence and absence of UTA1003, [opioid] is the concentration of UTA1003 used with DPDPE in the antagonism test [25,38].

### 4.4. Animal Maintenance and Care

Male Sprague Dawley (SD) rats (253.6 ± 3.9 g, 8 weeks old) obtained from the University of Tasmania animal services were housed as three littermates per cage at 22 °C with 50–60% humidity under an automated 12 h day/night cycle (lights on at 7:00 a.m.) with free access to food (Barastoc rodent cubes, Ridley Corporation, Melbourne, VIC, Australia) and water. Only male rats were used to avoid hormonal effects in female rats [116]. All procedures and handling were approved by the University of Tasmania Animal Ethics Committee (A0013864) and were conducted according to The Australian Code for the Care and Use of Animals for Scientific Purposes [117]. The experiments were conducted in compliance with the ARRIVE guidelines [118].

### 4.5. Preclinical Treatment Protocol

Animal body weights were recorded daily, immediately before experiments, in order to determine the dosage for each rat. The dosage of UFP-505, UTA1003, and morphine were calculated based on individual body weights. All drugs were administered as daily subcutaneous injections between the left thigh and the spinal cord. All rats were randomly divided as previously described [119] into five subgroups (n = 6, each) (morphine 3 mg/kg b.i.d.); UFP-505 27.1 mg/kg acute administration; UTA1003 24.6 mg/kg b.i.d.; a combination of morphine (3 mg/kg b.i.d.); and UTA1003 (24.6 mg/kg b.i.d.); or vehicle (DMSO 10% in 0.9% sodium chloride solution). Opioids were administered twice daily (mornings and evenings) over a period of 8 days (except for UFP-505). UFP-505 were excluded from long-term testing due to their negligible antinociceptive in vivo effects on day 1. Behavioural measurements after repeated daily vehicle treatment were not conducted due to its limited antinociceptive and behavioural effects after acute administration. UFP-505 and UTA1003 doses were calculated to provide the same molar concentrations compared to 40 mg/kg morphine sulfate. The amount of injected DMSO was kept equal for all used solutions. Reduced laboratory illumination intensity prior to and during experiments minimised any potential discomfort to the animals.

### 4.6. Assessment of Antinociception

Nociceptive thresholds were determined using two independent assays (tail flick and hot plate) using commercially available equipment (Ugo Basile, Comerio, Italy) (Figure 9). Maximum exposure to the nociceptive thermal stimulus was 15 s for the tail flick and 20 s for the hot plate assay. The infrared intensity of the tail flick photocell was set to 30, whereas the temperature of the hot plate was set to 54 ± 0.5 °C. On the first treatment day, all animals were tested in both assays immediately prior to the vehicle or opioid administration to obtain basal measurements as well as values for 15, 30, 60, and 120 min post administration. On all other days, the rats were tested prior to and 15, 30 min post injection. Nociception measurements were conducted in a blinded manner, and the mean of three independent measurements for each time point with a 1 min interval between measurements was recorded to minimise the ‘handling’ effects. The maximum possible effect (MPE) was defined as MPE % or antinociception = 100 × [(test latency − baseline latency)/(cut-off time − baseline latency)] as previously described [120]. The area under the curves (AUC) was calculated by the trapezoid method using GraphPad Prism V6 software (GraphPad Software Inc., La Jolla, CA, USA).

### 4.7. Behavioural Measurements

Behaviour was tested in the open-field arena of a fully automated Multi-Conditioning System (MCS, TSE GmbH, Homburg, Germany) that can assess and simultaneously analyse an extensive range of behavioural parameters of animals kept under controlled conditions (Figure 9). The MCS platform included an internal noise/light/temperature insulation system and a 3D infrared-beam frame that provided fast and accurate animal movement detection (100 Hz), combined with a high-resolution video monitoring and automated movement tracking system. Quantification and visualisation of the MCS data were processed by integrated system software (TSE ActiMot, TSE Systems, Chesterfield, MO, USA). The open-field arena was thoroughly cleaned and dried after testing each animal. A white background noise (10 dB) generator was used during all experiments in order to cancel out any unexpected laboratory sounds. On the 1st treatment day, the behaviour was assessed 1 min after nociception testing at all time points over a period of 5 min, while the rats were tested only 30 min post injection on the subsequent treatment days. Behavioural testing for this study included six different activity parameters (moving time, total distance travelled, rotation numbers, rearing numbers, rotation time, and rearing time). Rotation numbers were summarised from clockwise and counter-clockwise rotations as detected by the MCS. The area under the curves (AUC) for behavioural parameters were calculated by multiplication of behavioural effects (e.g., moving time) and treatment period (min or day). At the end of the observation period, animals were anaesthetised with 5% (*w*/*v*) isoflurane in oxygen at a flow rate of 1 L/min until the animal was unconscious (usually 5–7 min) before being decapitated.

### 4.8. Statistical Analysis

All data are expressed as mean ± SEM and analysed by two- or one-way ANOVA with Bonferroni’s multiple comparisons test, or unpaired *t*-test, using GraphPad Prism V6 software (GraphPad Software Inc., La Jolla, CA, USA). Multiple comparisons (Bonferroni’s test) were employed when F achieved *p* < 0.05, and there was no significant variance in homogeneity. A ‘*p*’ value less than 0.05 was considered statistically significant.

## Figures and Tables

**Table 1:** Summary of receptor specificity and efficacy of UTA1003 for the major opioid receptors.

Cells	EC_50_ of UTA1003	pEC_50_ of UTA1003	Emax (%)	Ref. Compound	EC_50_ of Ref. Compound	pEC_50_ of Reference	Emax (%)	Antagonist Effect (K_d_)
CHO-MOP	6.89 nM	8.16	82.44	DAMGO	12.5 nM	7.90	88.76	
CHO-DOP	26.6 nM	7.58	28.20	DPDPE	20.7 nM	7.68	68.18	47.8 nM
CHO-KOP	30.9 nM	7.51	94.47	U50,488	0.20 pM	12.70	95.94	
CHO-NOP	1.79 µM	5.75	28.34	Nociceptin	0.69 nM	9.16	90.73	

## Data Availability

Not applicable.

## Supplementary Materials

The supporting information can be downloaded at: https://www.mdpi.com/article/10.3390/ph15070789/s1, Figure S1: Antinociceptive effects (as latency in seconds) of different opioids after acute treatment; Figure S2: Antinociceptive effects (as latency in seconds) of chronic administration of opioids; Section S1: Synthesis of UFP-505 and novel UTA analogues.
