# Supplementary material for: Differential Effects of a Novel Opioid Ligand UTA1003 on Antinociceptive Tolerance and Motor Behaviour
Source: Pharmaceuticals (Basel). 2022 Jun 24;15(7):789. doi: 10.3390/ph15070789 (PMC9318816; doi:10.3390/ph15070789)
Supplement: Supplementary file 1 [file pharmaceuticals-15-00789-s001.zip › pharmaceuticals-1707712-supplementary.pdf]

pharmaceuticals

Supplementary Information

Differential effects of a novel opioid ligand UTA1003 on antinociceptive tolerance and motor behaviour

Alok K. Paul[1], Krystel L. Woolley[2], Mohammed Rahmatullah[3], Polrat Wilairatana[4*], Jason A. Smith[2], Nuri Gueven[1] and Nikolas Dietis[5*]

1 School of Pharmacy and Pharmacology, University of Tasmania, Hobart, TAS 7001, Australia; alok.paul@utas.edu.au (A.K.P.), nuri.guven@utas.edu.au (N.G.)
2 School of Natural Sciences, University of Tasmania, Hobart, TAS 7001, Australia
3 Department of Biotechnology & Genetic Engineering, University of Development Alternative, Lalmatia, Dhaka 1207, Bangladesh; rahmatm@uoda.edu.bd (M.R.)
4 Department of Clinical Tropical Medicine, Faculty of Tropical Medicine, Mahidol University, Bangkok 10400, Thailand; polrat.wil@mahidol.ac.th (P.W.)
5 Medical School, University of Cyprus, Nicosia 1678, Cyprus; dietis.nikolas@ucy.ac.cy (ND).
* Correspondence: dietis.nikolas@ucy.ac.cy (N.D.); polrat.wil@mahidol.ac.th (P.W.)

Antinociceptive Effects of UTA1003 after acute administration

A

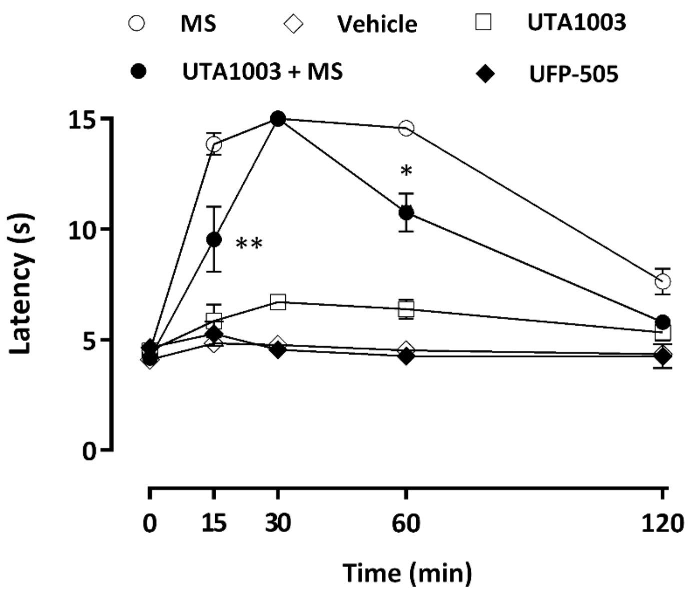

B

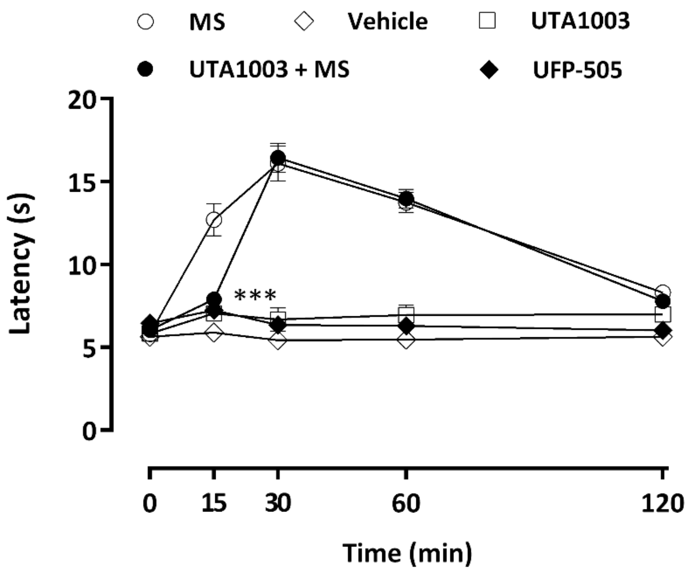

Supplementary Figure S1. Antinociceptive effects (as latency in seconds) of different opioids after acute treatment. Antinociceptive effects after single subcutaneous injections of UFP-505 (27.1 mg/kg), UTA1003 (24.6 mg/kg), morphine (MS, 3.0 mg/kg) or vehicle were measured in Sprague Dawley rats. Antinociception (as latency in seconds) was measured over a period of 120 min using tail flick (A) and hot plate (B) assays. Statistical significance against the effect of MS is shown as * $p < 0.05$, ** $p < 0.01$ and *** $p < 0.001$ for the same time-point and was calculated using one-way ANOVA with Bonferroni's multiple comparisons. Values are presented as Mean ± SEM (n = 6 animals per group).

Antinociceptive Effects of UTA1003 after chronic administration

A

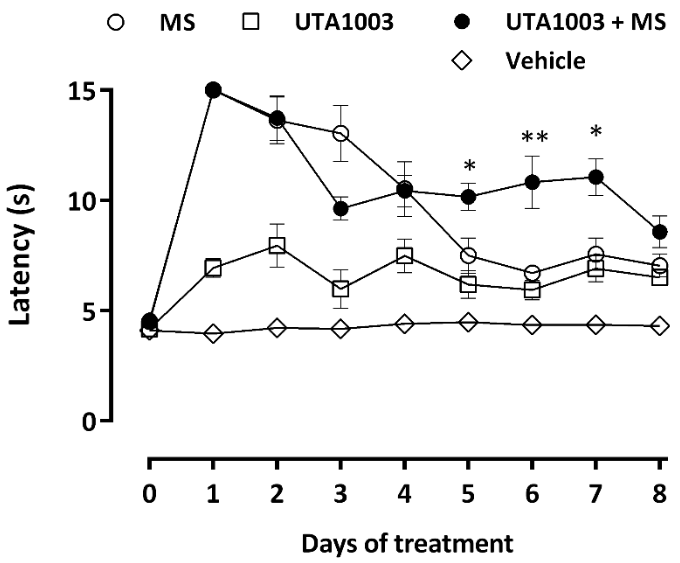

B

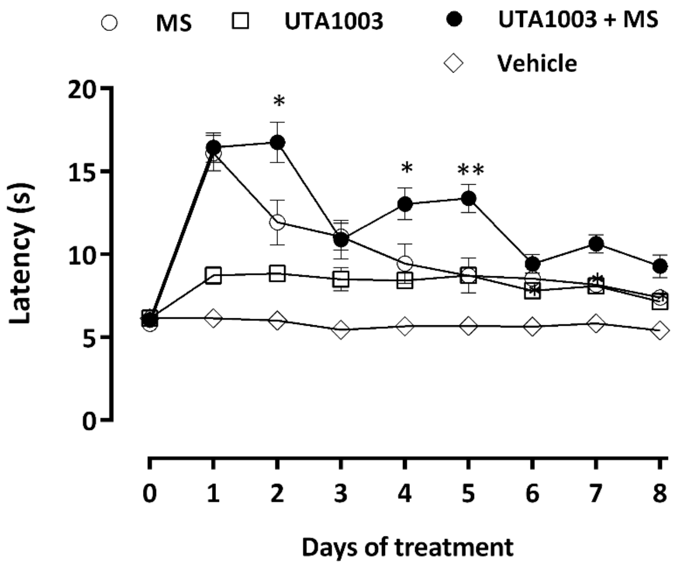

Supplementary Figure S2. Antinociceptive effects (as latency in seconds) of chronic administration of opioids. Antinociceptive effects after twice-daily subcutaneous treatment with vehicle, UTA1003 (24.6 mg/kg), morphine (MS, 3 mg/kg) or their combination were measured in Sprague Dawley rats. Antinociception (as latency in seconds) was measured daily at 30 min post injections over a period of 8 days using tail-flick (A) and hot-plate (B) assays. Statistically significant differences in antinociception at the same time-point were calculated between MS and MS+UTA1003 treated animals. Statistical significance was calculated using One-way ANOVA with Bonferroni's multiple comparisons and shown as $^{*}p < 0.05$ and $^{**}p<0.01$. Values are presented as mean ± SEM (n = 6 animals per group).

Appenxdix 1. Synthesis of UFP-505 and novel UTA analogues

UFP-505 was synthesised by the method previously reported by Balboni and colleagues [1]. Novel compounds UTA1003, UTA1005, UTA1009 and UTA1011 were synthesised similarly to the method for UFP-505 and details are provided in the supplementary information. by Products were analysed by mass spectrometry.

General Procedure for Peptide Coupling and Deprotection of UFP-505 and analogues

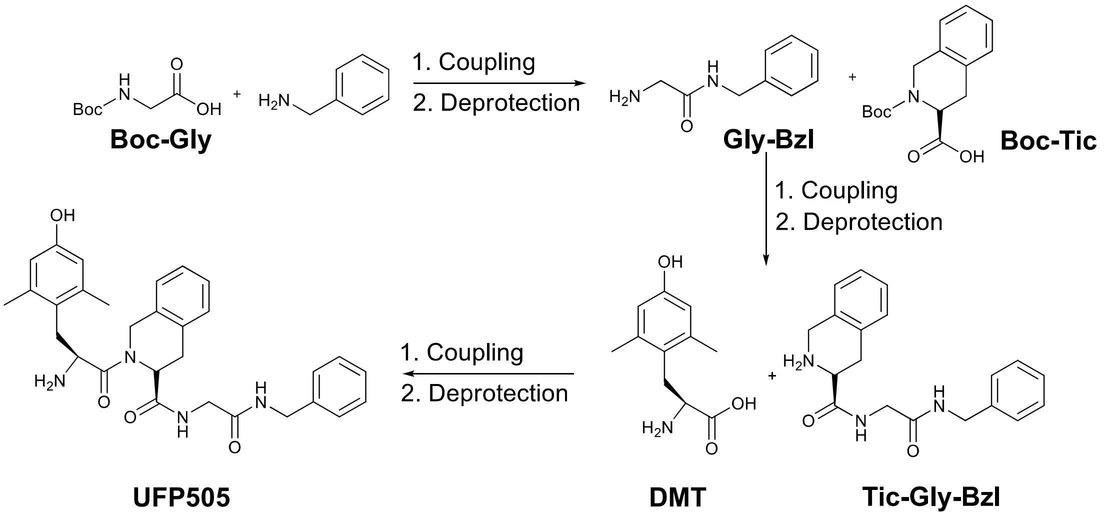

Peptide Coupling

Amino acid (1 equiv.) was added to anhydrous DMF under an atmosphere of N_2 and was cooled to 0 °C. Amine (1 equiv.), DMAP (0.1 equiv.), Et_3N (2.5 equiv.) and a coupling agent (1.4 equiv.) were added and the reaction mixture warmed slowly to room temperature before leaving overnight. The reaction was quenched with sat. $NaHCO_3$ (20mL) and the organic layer washed with sat. K_2SO_4 solution, sat. $NaHCO_3$ solution and H_2O. The organic layer was dried with $MgSO_4$, filtered and the solvent removed under reduced pressure to give a clear crude oil which was redissolved in *n*-hexanes (5.0 mL) and diethyl ether added slowly until a precipitate was formed. The solvent was decanted and evaporated under reduced pressure to give the Boc-protected product.

Peptide Deprotection

The product from the coupling reaction was added to TFA (5.0 mL) and the reaction mixture left at room temperature for 30 min with constant stirring. TFA was removed under reduced pressure to give a clear crude oil which was redissolved in *n*-hexanes (5.0 mL) and diethyl ether added slowly until a precipitate was formed. The solvent was decanted and evaporated under reduced pressure to give the TFA salt.

Boc-Gly-Bzl /TFA-Gly-Bzl

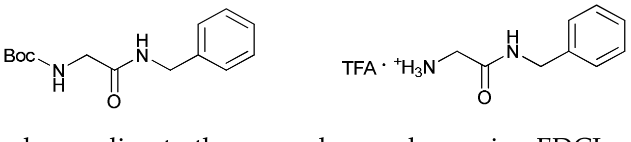

Boc-Gly-Bzl was prepared according to the general procedure using EDCI as the coupling agent and CH_2Cl_2 as the solvent using Boc-Gly-OH (10.02 g, 57.22 mmol) and benzylamine (6.3 mL, 57.68 mmol) to give the **Boc-Gly-Bzl** as a white solid in 48 % yield (7.340 g, 27.73 mmol). Removal of the Boc protecting group was performed according to the general procedure to give **TFA-Gly-Bzl** as a white solid in 93 % yield (7.209 g, 25.91 mmol).

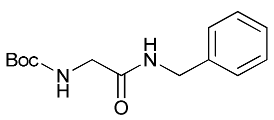

^{1}H NMR δ ($CDCl_3$, 400 MHz): 1.55 (s, 9H), 3.74 (bd, *J* = 3 Hz, 2H), 4.33 (bd, *J* = 6.0 Hz, 2H), 5.68 (bs, 1H), 7.17-7.23 (m, 5H)

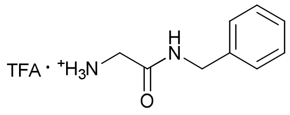

^{1}H NMR δ ($CDCl_3$, 400 MHz): 3.42 (s, 2H), 4.10 (d, *J* = 5.7 Hz, 2H), 6.93 - 7.01 (m, 5H), 8.16 (bs, 3H), 8.48 (t, *J* = 5.4 Hz, 1H); ^{13}C NMR δ ($CDCl_3$, 100 MHz) 30.3, 42.7, 126.7, 127.1, 127.9, 165.6

Boc-Tic-Gly-Bzl/ TFA-Tic-Gly-Bzl

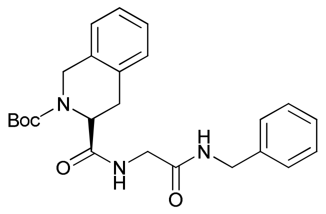

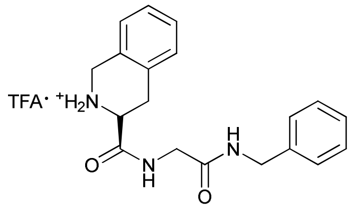

Boc-Tic-Gly-Bzl was prepared according to the general procedure using BOP as the coupling agent starting from Boc-Tic-OH (108.1 mg, 0.3898 mmol) and **TFA·Gly-Bzl** (99.4 mg, 0.3573 mmol) to give the title compound as a white solid in 89 % yield (135.9 mg, 0.3209 mmol). Removal of the Boc protecting group was performed according to the general procedure to give **TFA-Tic-Gly-Bzl** as a white solid in 81 % yield (117.4 mg, 0.2684 mmol).

^{1}H NMR δ ($CDCl_3$, 400 MHz): 2.91 – 3.04 (m, 2H), 3.50 (dd, *J* = 17.0, 3.6 Hz, 1H), 3.98 – 4.03 (m, 2H), 4.08 – 4.13 (m, 3H), 4.23 (dd, *J* = 14.6, 5.9 Hz, 1H), 6.87 – 7.10 (m, 9H), 8.01 (s, 1H), 8.81 (s, 1H); ^{13}C NMR δ ($CDCl_3$, 100 MHz): 29.2, 42.4, 42.8, 43.8, 54.5, 126.0, 126.5, 126.9, 127.4, 127.5, 127.8, 128.3, 130.4, 138.8, 168.5, 168.6 (one carbon overlapping or missing)

Boc-DMT-Tic-Gly-Bzl / H-DMT-Tic-Gly-Bzl (UFP505)

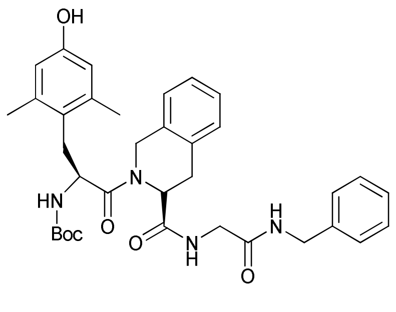

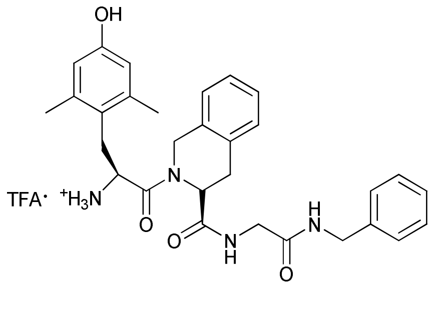

Boc-DMT-Tic-Gly-Bzl was prepared according to the general procedure using PyBOP as the coupling agent with Boc-DMT-OH (30.9 mg, 0.0999 mmol) and **TFA-Tic-Gly-Bzl** (50.9 mg, 0.1163 mmol) to give the titled crude product as a white solid in 88 % yield (54.2 mg, 0.0880 mmol). Removal of the Boc protecting group was performed according to the general procedure to give **TFA-DMT-Tic-Gly-Bzl** as a white solid in 85 % yield (47.3 mg, 0.0752 mmol).

MS *m*/*z* (ES+) 515 (M+H, 100), 537 (M+Na, 10)

Synthesis of UTA1003

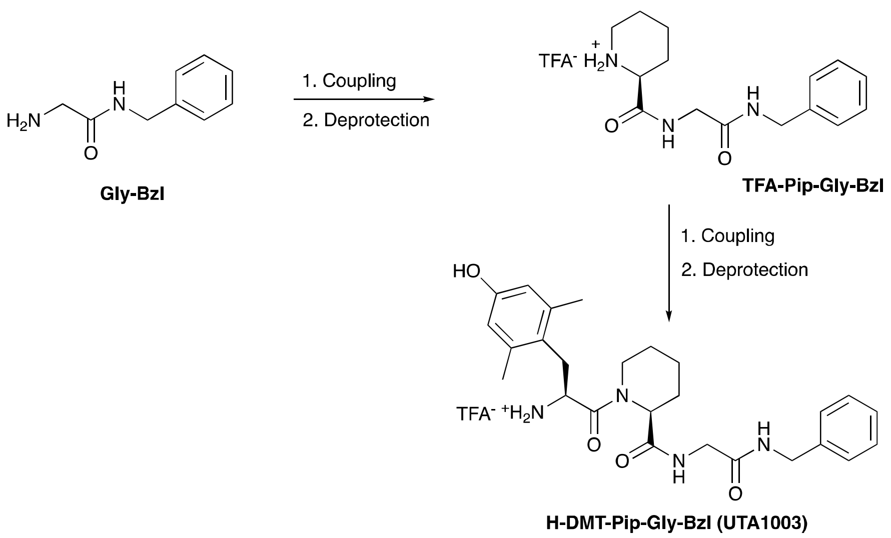

Boc-Pip-Gly-Bzl/ TFA-Pip-Gly-Bzl

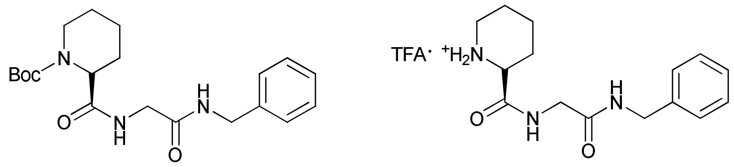

Boc-Pip-Gly-Bzl was prepared according to the general procedure using BOP as the coupling agent with Boc-Pip-OH (202.9 mg, 0.8850mmol) and **TFA-Gly-Bzl** (247.4 mg, 0.8892 mmol) to give the titled crude product as a white solid in 91 % yield (299.0 mg, 0.7936 mmol). Removal of the Boc protecting group was performed according to the general procedure to give **TFA-Pip-Gly-Bzl** as a white solid in a crude yield of >95 % yield (310.8 mg, 0.7982 mmol).

^{1}H NMR δ ($CDCl_3$, 400 MHz): 1.00 – 1.18 (m, 2H), 1.39 – 1.50 (m, 2H), 2.38 – 1.53 (m, 2H), 2.90 (d, *J* = 12.6 Hz, 1H), 3.28 (dd, *J* = 16.3, 5 Hz, 1H), 3.45 (d, *J* = 12.5 Hz, 1H), 3.68 (dd, *J* = 16.8, 6.9 Hz, 1H), 3.88 (dd, *J* = 13.8, 5.6 Hz, 1H), 3.94 - 4.01 (m, 2H), 6.76 – 6.87 (m, 5H), 7.89 (t, *J* = 6.4 Hz, 1H), 8.37 (t, *J* = 5.1 Hz, 1H), 8.44 (t, *J* = 5.1 Hz, 1H)

Boc-DMT-Pip-Gly-Bzl/ H-DMT-Pip-Gly-Bzl (UTA1003)

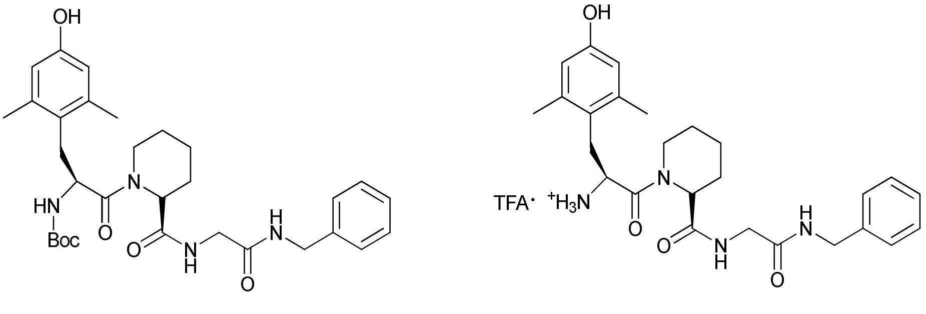

Boc-DMT-Pip-Gly-Bzl was prepared according to the general procedure using PyBOP as the coupling agent with Boc-DMT-OH (100.8 mg, 0.3258 mmol) and **TFA-Pip-Gly-Bzl** (127.8 mg, 0.3282 mmol) to give the titled crude product as a white solid in 68 % yield (125.3 mg, 0.2211 mmol). Removal of the Boc protecting group was performed according to the general procedure to give **TFA-DMT-Pip-Gly-Bzl** as a white solid in 96 % yield (123.2 mg).

MS *m/z* (ES+) 467 (M+H, 100)

Synthesis of UTA1005

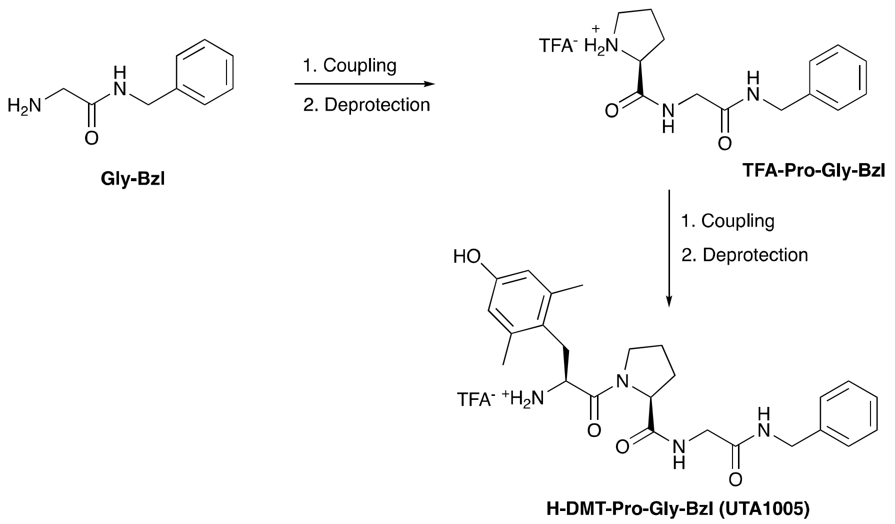

Boc-Pro-Gly-Bzl/ TFA-Pro-Gly-Bzl

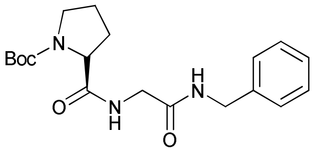
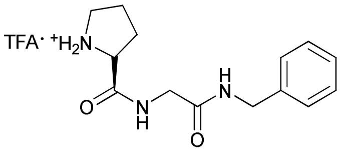

Boc-Pro-Gly-Bzl was prepared according to the general procedure using BOP as the coupling agent with Boc-Pro-OH (445.4 mg, 1.601 mmol) and **TFA-Gly-Bzl** (343.3 mg, 1.595 mmol) to give the title compound a white solid in 93 % yield (536.4mg). Removal of the Boc protecting group was performed according to the general procedure to give **TFA-Pro-Gly-Bzl** as a white solid in >95 % yield.
^{1}H NMR δ ($CDCl_3$, 400 MHz): 1.93 – 2.00 (m, 3H), 2.30 – 2.35 (m, 1H), 3.24 – 3.36 (m, 2H), 3.74 (dd, *J* = 16.0, 5.7 Hz, 1H), 4.16 (dd, *J* = 16.6, 6.8 Hz, 1H), 4.33 (dd, *J* = 14.9, 5.7 Hz, 1H), 4.39 – 4.45 (m, 2H); ^{13}C NMR δ ($CDCl_3$, 100 MHz): 24.3, 34.8, 43.1, 43.4, 46.3, 59.7, 127.5, 128.6, 128.7, 137.7, 169.1, 169.3

Boc-DMT-Pro-Gly-Bzl/ H-DMT-Pro-Gly-Bzl (UTA1005)

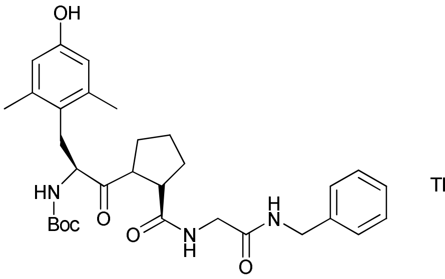
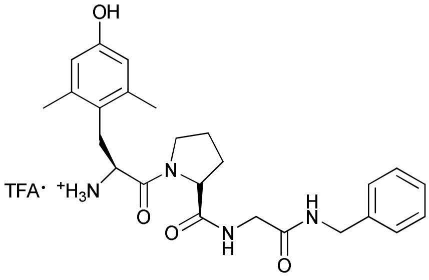

Boc-DMT-Pro-Gly-Bzl was prepared according to the general procedure using PyBOP as the coupling agent with Boc-DMT-OH (101.4 mg, 0.3278 mmol) and **TFA-Pro-Gly-Bzl** (124.7 mg, 0.3322 mmol) to give the titled crude product as a white solid in 79 % yield (142.5 mg, 0.2583 mmol). Removal of the Boc protecting group was performed according to the general procedure to give **TFA-DMT-Pro-Gly-Bzl** as a white solid in 86 % yield (126.4 mg).
MS *m/z* (ES+) 453 (M+H, 100), 475 (M+ Na, 5)

Synthesis of UTA1009

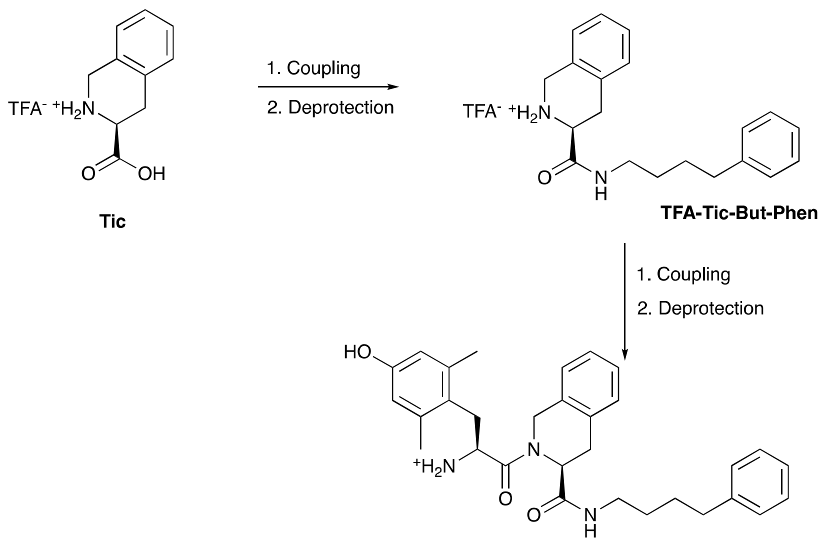

H-DMT-Tic-But-Phen (UTA1009)

Boc-Tic-But-Phen/ TFA·Tic-But-Phen

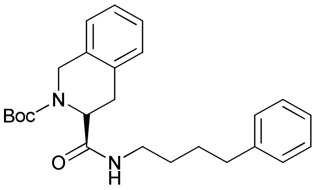

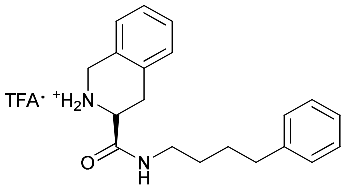

Boc-Tic-But-Phen was prepared according to the general procedure using BOP as the coupling agent with Boc-Tic-OH (499.7 mg, 1.802 mmol) and 4-phenylbutylamine (0.29 ml, 1.834 mmol) to give the title compound as a crude white solid in 89 % yield (657.1 mg, 1.608 mmol). Removal of the Boc protecting group was performed according to the general procedure to give **TFA-Tic-But-Phen** as a white solid in 97 % yield (661.0 mg, 1.565 mmol).

^{1}H NMR δ (DMSO-D6, 400 MHz): 1.47 (quin, *J* = 7.4 Hz, 2H), 1.61 (quin, *J* = 7.4 Hz, 2H), 2.60 (t, *J* = 7.6 Hz, 2H), 2.96 (dd, *J* = 15.9, 12.1 Hz, 1H), 3.17 - 3.30 (m, 3H), 4.10 (dd, *J* = 12.0, 4.2 Hz, 1H), 4.32 (q, *J* = 16.1 Hz, 2H), 7.15 – 7.30 (m, 9H), 8.52 (s, 1H), 9.43 (s, 2H); ^{13}C NMR δ (DMSO-D6, 100 MHz): 28.1, 28.5, 29.4, 34.7, 38.5, 43.9, 54.1, 125.7, 126.5, 126.9, 127.5, 128.25, 128.29, 128.4, 128.6, 130.8, 141.9, 167.6

Boc-DMT-Tic-But-Phen/ TFA-DMT-Tic-But-Phen (UTA1009)

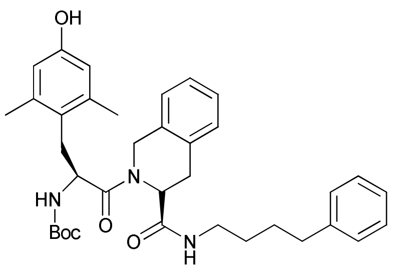

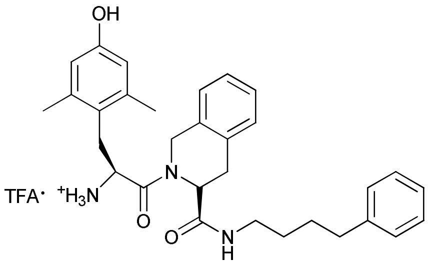

Boc-DMT-Tic-But-Phen was prepared according to the general procedure using PyBOP as the coupling agent with Boc-DMT-OH (101.2 mg, 0.3271 mmol) and **TFA-Tic-But-Phen** (137.0 mg, 0.3243 mmol) to give the titled crude product as a white solid in 79 % yield (155.5 mg, 0.2593 mmol). Removal of the Boc protecting group was performed according to the general procedure to give **TFA-DMT-Tic-But-Phen** as a white solid in a 93 % yield (147.8 mg, 0.2408 mmol).

MS *m/z* (ES+) 500 (M+H, 100), 522 (M+Na, 10)

Synthesis of UTA1011

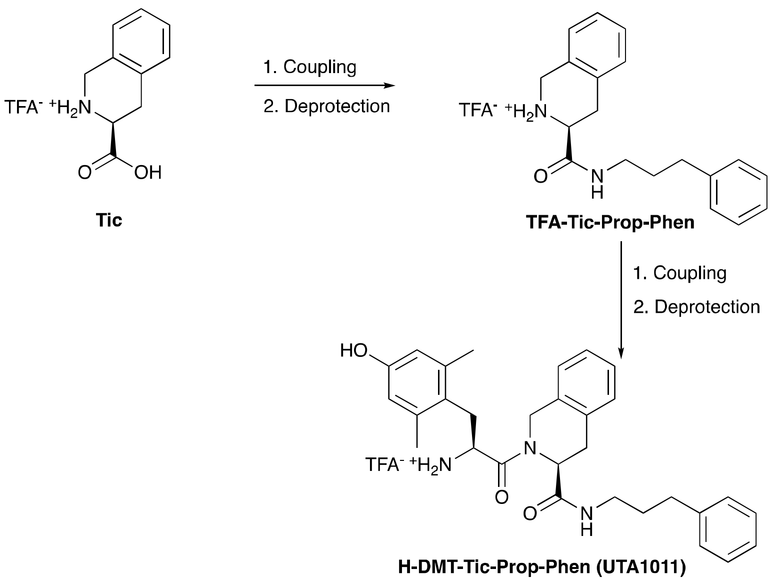

Boc-Tic-Prop-Phen/ TFA-Tic-Prop-Phen

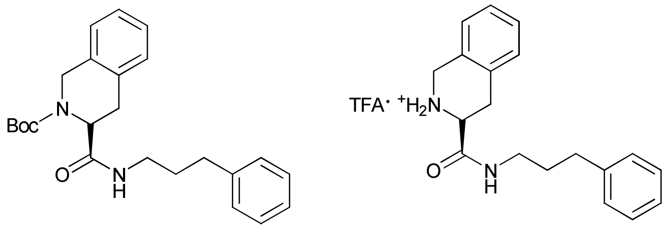

Boc-Tic-Prop-Phen was prepared according to the general procedure using BOP as the coupling agent with Boc-Tic-OH (202.1 mg, 0.7287 mmol) and 3-phenylpropylamine (1.0 mL, 0.7777 mmol) to give the titled crude product as a white solid in >95 % yield (321.3 mg, 0.8144 mmol). Removal of the Boc protecting group was performed according to the general procedure to give **TFA·Tic-Prop-Phen** as a white solid in a >95 % yield (294.1 mg, 0.7201 mmol).

^{1}H NMR δ (DMSO-D6, 400 MHz): 1.81 – 1.88 (m, 2H), 2.65 – 2.74 (m, 2H), 3.18 – 3.23 (m, 2H), 3.30 - 3.38 (m, 2H), 4.47 (d, *J* = 15.4 Hz, 1H), 4.66 (d, *J* = 15.4 Hz, 1H), 4.74 (t, *J* = 8.5 Hz, 1H), 6.84 (bs, 1H), 6.99 – 7.23 (m, 9H), 8.16 (bs, 1H); ^{13}C NMR δ (DMSO-D6, 100 MHz): 29.9, 31.0, 32.9, 39.1, 43.9, 54.7, 125.9, 126.4, 127.2, 127.9, 128.3, 128.4, 128.6, 130.7, 141.5, 141.6, 168.5,

Boc-DMT-Tic-Prop-Phen/ H-DMT-Tic-Prop-Phen (UTA1011)

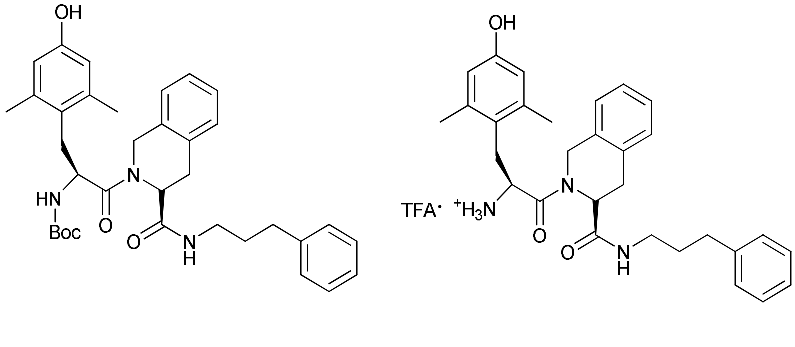

Boc-DMT-Tic-Prop-Phen was prepared according to the general procedure using PyBOP as the coupling agent with Boc-DMT-OH (100.3 mg, 0.3242 mmol) and **TFA-Tic-Prop-Phen** (131.5 mg, 0.3219 mmol) to give the titled crude product as a white solid in 79 % yield (150.6 mg, 0.2562 mmol). Removal of the Boc protecting group was performed according to the general procedure to give **TFA-DMT-Tic-Prop-Phen** as a white solid in quantitative yield (165.4 mg, 0.2758 mmol).

MS *m/z* (ES+) 486 (M+H, 30), 508 (M+Na, 8)

Reference:

1. Balboni, G.; Guerrini, R.; Salvadori, S.; Bianchi, C.; Rizzi, D.; Bryant, S.D.; Lazarus, L.H. Evaluation of the dmt-tic pharmacophore: Conversion of a potent delta-opioid receptor antagonist into a potent delta agonist and ligands with mixed properties. *J Med Chem* **2002**, *45*, 713-720.
